# Pharmacological thromboprophylaxis to prevent venous thromboembolism in patients with temporary lower limb immobilization after injury: systematic review and network meta‐analysis

**DOI:** 10.1111/jth.14666

**Published:** 2019-12-01

**Authors:** Daniel Horner, John W. Stevens, Abdullah Pandor, Tim Nokes, Jonathan Keenan, Kerstin de Wit, Steve Goodacre

**Affiliations:** ^1^ Emergency Department Salford Royal NHS Foundation Trust Salford UK; ^2^ School OF Health and Related Research The University of Sheffield Sheffield UK; ^3^ University Hospitals Plymouth NHS Trust Plymouth UK; ^4^ Department of Medicine McMaster University Hamilton ON Canada

**Keywords:** risk, immobilization, venous thromboembolism, casts, surgical, network meta‐analysis

## Abstract

**Background:**

Thromboprophylaxis has the potential to reduce venous thromboembolism (VTE) following lower limb immobilization resulting from injury.

**Objectives:**

We aimed to estimate the effectiveness of thromboprophylaxis, compare different agents, and identify any factors associated with effectiveness.

**Methods:**

We undertook a systematic review and network meta‐analysis (NMA) of randomized trials reporting VTE or bleeding outcomes that compared thromboprophylactic agents with each other or to no pharmacological prophylaxis, for this indication. An NMA was undertaken for each outcome or agent used, and a series of study‐level network meta‐regressions examined whether population characteristics, type of injury, treatment of injury, or duration of thromboprophylaxis were associated with treatment effect.

**Results:**

Data from 6857 participants across 13 randomized trials showed that, compared with no treatment, low molecular weight heparin (LMWH) reduced the risk of any VTE (odds ratio [OR]: 0.52; 95% credible interval [CrI]: 0.37‐0.71), clinically detected deep vein thrombosis (DVT) (OR: 0.39; 95% CrI: 0.12‐0.94) and pulmonary embolism (PE) (OR: 0.16; 95% CrI: 0.01‐0.74), whereas fondaparinux reduced the risk of any VTE (OR: 0.13; 95% CrI: 0.05‐0.30) and clinically detected DVT (OR: 0.10; 95% CrI: 0.01‐0.86), with inconclusive results for PE (OR: 0.40; 95% CrI: 0.01‐7.53).

**Conclusions:**

Thromboprophylaxis with either fondaparinux or LMWH appears to reduce the odds of both asymptomatic and clinically detected VTE in people with temporary lower limb immobilization following an injury. Treatment effects vary by outcome and are not always conclusive. We were unable to identify any treatment effect modifiers other than thromboprophylactic agent used.

**Essentials**

- Patients with injury and lower limb immobilization are at increased risk of thromboembolism.
- This network meta‐analysis analyzed 6857 patients randomized to thromboprophylaxis or control.
- Overall, pharmacological prophylaxis significantly reduced the odds of any venous thromboembolism (VTE).
- Individualized treatment may be an optimal strategy and requires further study.

## 1 BACKGROUND

Venous thromboembolic (VTE) disease is a major global cause of morbidity and mortality.^1^, ^2^ An estimated 10 million episodes are diagnosed yearly; more than one‐half of these episodes are provoked by hospital admission/procedures and result in significant loss of disability‐adjusted life years.^3^

Temporary lower limb immobilization after injury is a significant contributor to overall VTE burden.^4^ This risk may be modifiable. Existing evidence suggests that pharmacological prophylaxis could reduce overall VTE event rates in these patients, but the proportional reduction of symptomatic events remains unclear.^5^ Recent randomized controlled trials (RCTs) have used different pharmacological agents (low molecular weight heparin [LMWH] and fondaparinux), dosing regimens and outcome measures.^6^, ^7^, ^8^, ^9^ In addition, some centers are reporting recent experience with use of the direct oral anticoagulants (DOACs) for this indication, despite the lack of appropriate licensing and trial data within this specific population.^10^, ^11^

Consequently, there is wide variation in thromboprophylaxis strategies, and international guidelines continue to offer conflicting advice for clinicians.^12^, ^13^, ^14^, ^15^ The overall clinical effectiveness of thromboprophylaxis for this indication and the optimal agent/dosing strategy are yet to be defined.

We undertook a systematic review and network meta‐analysis (NMA) to assess the effectiveness of pharmacological thromboprophylaxis at preventing VTE in patients with temporary lower limb immobilization after injury. Our aim was to estimate the clinical effectiveness for each pharmacological thromboprophylaxis option and further compare regimens and agents to identify an optimal strategy.

## 2 METHODS

The systematic review was undertaken in accordance with the general principles recommended in the Preferred Reporting Items for Systematic Reviews and Meta‐Analyses (PRISMA) statement.^16^ This review was part of a larger project on thromboprophylaxis for lower limb immobilization that was registered on the PROSPERO international prospective register of systematic reviews (CRD42017058688).^17^ The full protocol is available here (https://www.journalslibrary.nihr.ac.uk/programmes/hta/1518706/#/).

### 2.1 Eligibility criteria

Studies were considered eligible for inclusion if they met the following criteria: (a) RCTs or controlled clinical trials; (b) adults (age >16 years) requiring temporary immobilization (e.g., leg cast or brace in an ambulatory setting) for an isolated lower limb injury; (c) chemical thromboprophylaxis with any LMWH agent, fondaparinux, or oral anticoagulant (e.g., apixaban, dabigatran etexilate, rivaroxaban, edoxaban); (d) comparators included placebo, no treatment, aspirin, or alternative treatment; and (e) outcomes included symptomatic or asymptomatic deep vein thrombosis, pulmonary embolism (PE), major bleeding (as defined within each study), or mortality. Exclusion criteria for selection included studies that had not been designed as experimental studies (e.g., cohort studies, case control studies); involved hospital inpatient care or any patient requiring hospital admission longer than 3 days; patients receiving mechanical thromboprophylaxis or undergoing ambulant orthopedic surgery (e.g., arthroscopy, arthroscopic surgery).

### 2.2 Outcome definitions

Given the challenges of outcome reporting in this population, we chose to prospectively define VTE events according to anatomical location and symptomatology. Our aim was to provide full transparency of all potentially relevant outcomes and to highlight the specific data informing assessment of intervention.

We defined proximal deep vein thrombosis (DVT) as thrombosis occurring at or above the level of the popliteal trifurcation. 18 Symptomatic disease was defined as reported within individual trials; any diagnosis of PE was considered to be symptomatic, as were presentations outside routine study follow‐up with acute DVT symptoms and subsequent confirmation of disease. However, in several studies patients were questioned on the symptoms of DVT (e.g., pain, swelling) when the cast was removed, at routine follow‐up. If the patients reported any positive symptoms and routine sonography had detected DVT, the event was classified as symptomatic. The limitations with this approach are highlighted later in the discussion section. “Any VTE”was defined as the composite of any PE and/or any distal or proximal DVT, with or without symptoms.

We could not retrospectively apply consensus definitions of symptomatology or major bleeding to individual study results.^19^ These issues and their potential impact on study results are explored further in the discussion section.

### 2.3 Information sources and searches

Ten electronic databases (including MEDLINE, EMBASE, and the Cochrane Library) were searched. The search strategy used free text and thesaurus terms and combined synonyms relating to the condition (e.g., venous thromboembolism in people with lower limb immobilization) with synonyms relating to the interventions (e.g., LMWH, aspirin, oral anticoagulants). No language restrictions were used. Searches were supplemented by hand‐searching the reference lists of all relevant studies (including existing systematic reviews), performing a citation search of relevant articles, contacting key experts in the field, and undertaking systematic keyword searches of the World Wide Web using the Google search engine. Further details on the search strategy can be found in Table S1 (supporting information).

### 2.4 Study selection

All titles were examined for inclusion by one reviewer (A.P.); any citations that clearly did not meet the inclusion criteria were excluded. All abstracts and full‐text articles were then examined independently by two reviewers (A.P. and D.H.). Any disagreements in the selection process were resolved through discussion or if necessary, arbitration by a third reviewer (S.G.) and included by consensus.

### 2.5 Data extraction and quality assessment

Data relating to study design, methodological quality, and outcomes were extracted by one reviewer into a standardized data extraction form and independently checked for accuracy by a second.

The methodological quality of each included study was evaluated using a revised Cochrane Risk of Bias tool for randomized trials (RoB 2.0).^20^ The original tool 21 was updated because of questionable inter‐rater agreement, subjectivity in assigning risk of bias judgments, and bias judgments assigned at the trial level.^22^, ^23^, ^24^, ^25^ An overall judgement of bias was assigned as low risk if all domains were judged as low risk of bias; high risk if at least one domain was judged to be at high risk of bias (or if the study has some concerns for multiple domains in a way that substantially lowers confidence in the result), and some concerns if any bias (other than high risk) was noted in at least one domain.^20^

### 2.6 Data synthesis and analysis

For each outcome of interest, an NMA was performed to allow a simultaneous comparison between interventions using all available studies. The data were the number of events out of the number of patients randomized to each class of intervention, which were assumed to arise from an underlying binomial distribution. LMWH agents were collated and considered as a single intervention. The probabilities of an event for each intervention were modelled using a logistic model to estimate odds ratios (ORs). The reference intervention was defined as placebo, no treatment, or aspirin in the NMA. The different thromboprophylaxis drugs were treated as separate interventions (i.e., LMWH, DOACs, and fondaparinux) in the NMA on the basis of having different mechanisms of action and different adverse event profiles.

The analysis was implemented using Markov chain Monte Carlo simulation using WinBUGS software Version 1.4.3 (MRC Biostatistics Unit).^26^ A fixed effect model was used to estimate the effects of LMWH and fondaparinux relative to control in the available studies (i.e., a conditional inference). In addition, a random effects model was used to allow for heterogeneity in the effects of interventions between studies and to estimate whether the interventions can have an effect in future studies. Results were presented using ORs, 95% credible intervals (CrI), and 95% predictive intervals for the OR in a randomly chosen study relative to the control, with the probability of each intervention being the best.

We also evaluated the following potential treatment effect modifiers in a series of meta‐regressions: (a) Population characteristics (proportion male, baseline risk of VTE); (b) type of injury (fractures, Achilles tendon rupture, other soft‐tissue injury); (c) treatment of injury (surgical versus conservative, above versus below knee immobilization); (d) thromboprophylactic agent used; and (5) duration of thromboprophylaxis.

## 3 RESULTS

### 3.1 Study selection

**Figure 1:**
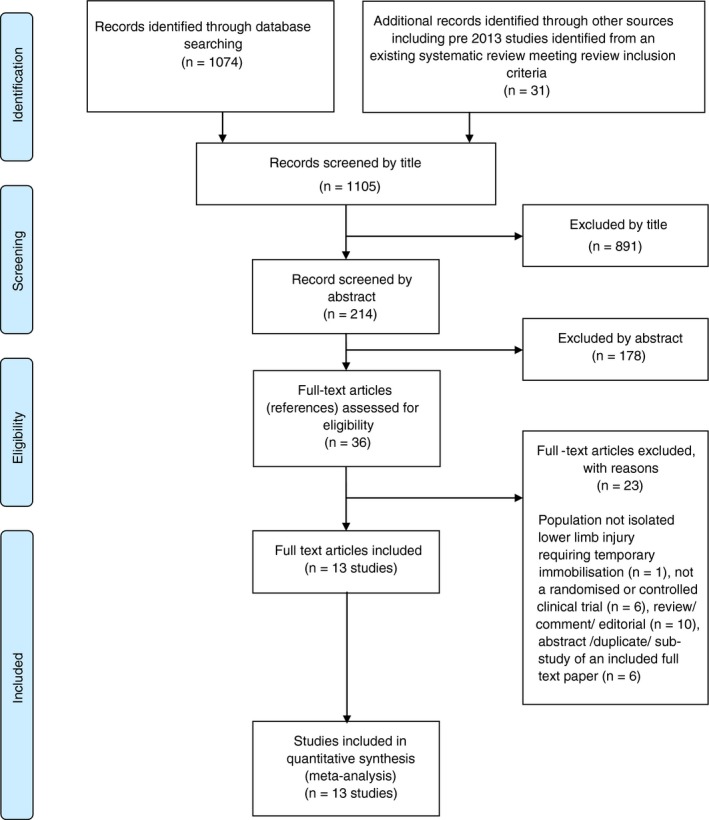
Study flow chart (adapted)

The literature searches identified 1105 citations. Of these, 13 RCTs met the inclusion criteria.^6^, ^8^, ^9^, ^27^, ^28^, ^29^, ^30^, ^31^, ^32^, ^33^, ^34^, ^35^, ^36^ A flow chart describing the process of identifying relevant literature can be found in Figure 1. Studies excluded after full text review are listed in Table S2, along with the rationale for exclusion.

### 3.2 Characteristics of included studies

The design and patient characteristics of the 13 included studies^6^, ^8^, ^9^, ^27^, ^28^, ^29^, ^30^, ^31^, ^32^, ^33^, ^34^, ^35^, ^36^ are summarized in Table 1. All studies were published between 1993 and 2017. In total, 6857 patients were included and randomized across 10 different countries (Canada,^8^, ^28^ China,^6^ Denmark,^29^, ^34^ France,^35^ Germany,^27^, ^30^, ^31^, ^35^ Italy,^35^ Netherlands,^9^, ^35^, ^36^ Russia,^35^ Spain^35^ and Sweden^32^, ^33^) to receive either intervention or control. LMWH injections were the primary intervention, using variable agents (certoparin,^30^ dalteparin,^8^, ^28^, ^32^, ^33^ nadroparin,^9^, ^31^, ^35^, ^36^ reviparin,^27^, ^34^ and tinzaparin)^29^ and dosing regimens. Two studies used fondaparinux.^9^, ^35^ Eleven of the studies compared LMWH thromboprophylaxis to no thromboprophylaxis, one three‐arm study compared LMWH with fondaparinux with no thromboprophylaxis, and one study compared LMWH with fondaparinux. We found no randomized trials comparing DOACs with any other thromboprophylaxis strategy for this patient group. One study used aspirin as a control group,^27^ with others using placebo injections or nothing dependent on design.^6^, ^8^, ^28^, ^29^, ^30^, ^31^, ^32^, ^33^, ^34^, ^36^ In general, most studies excluded patients at highest risk of VTE (e.g., active cancer,^6^, ^8^, ^9^, ^32^, ^33^ previous VTE^6^, ^8^, ^9^, ^28^, ^30^, ^31^, ^32^, ^33^, ^34^, ^36^ or first‐degree family history of VTE^6^, ^36^).

**Table 1:** Summary of design and patient characteristics

Author year	Country (sites)	Design	Population	Time between injury and recruitment/ immobilization duration (mean)	Prophylaxis before randomization	Intervention	Comparator	Outcome measure (primary)
Goel et al.^28^	Canada (NR)	R, DB^a^	Adults (18‐75 y; mean age, 41 y; male, 62%). Fractures below knee. Surgically treated Outpatients N = 305	Within 48h Immobilization duration: 14 d^b^	No	LMWH (dalteparin, 5000 IU/d for 14 d; administered by s.c. injection; compliance, >95%)	Matching placebo for 14 d (compliance with injections, >95%)	Incidence of DVT determined by bilateral venography at end of treatment
Jørgensen et al.*,* 2002 29	Denmark (3 centers)	R, OL^a^	Adults (>18 y; mean age, 48 y; male, 57%) Fracture or soft‐tissue injury; Conservative or surgically treated Outpatients N = 300	NR Immobilization duration: 5.5 weeks	No	LMWH (tinzaparin, 3500 IU/d for duration of cast immobilization; administered by s.c. injection; compliance, NR)	No treatment	Incidence of DVT determined by unilateral venography after plaster cast removal
Kock et al., 1995^30^	Germany (NR)	R, OL	Adults (18‐75 y; mean age, 34 y; male, 61%) Fracture or soft‐tissue injury Conservative treatment Outpatients N = 428	NR Immobilization duration: 17 d^b^	No	LMWH (certoparin, 3000 IU/d for duration of cast immobilization; administered by s.c. injection; compliance, NR)	No treatment	Incidence of DVT determined by duplex sonography and confirmed by phlebography after plaster cast removal
Kujath et al.*,* 1993 31	Germany (1 hospital)	R, OL	Patients over 16 y (mean age, 34 y; male, 58%) Fracture or soft‐tissue injury Conservative treatment Outpatients N = 306	NR Immobilization duration: 15.7 d^b^	No	LMWH (Nadroparin, 2850 IU/d for duration of cast immobilization; administered by s.c. injection; compliance, NR)	No treatment	Incidence of DVT determined by compression ultrasound and phlebography (positive findings only) after plaster cast removal
Lapidus et al.*,* 2007a 32	Sweden (1 centra)	R, DB^a^	Adults (18‐75 y; mean age, 40 y; male, 79%) Soft‐tissue injury (Achilles tendon rupture) Surgically treated Outpatients N = 105	Within 72 h of injury Immobilization duration: 43 d^b^	No	LMWH (dalteparin, 5000 IU/d for 6 weeks; administered by s.c. injection; compliance, NR)	Matching placebo for 6 weeks (compliance with injections, NR)	Incidence of DVT determined by unilateral duplex sonography and confirmed by phlebography
Lapidus et al.*,* 2007b 33	Sweden (1 centra)	R, DB^a^	Adults (18‐75 y; mean age, 48 y; male, 46%) Fracture of the ankle Surgically treated Outpatients N = 272	Within 72 h of injury Immobilization duration: 44 d^b^	Yes, all patients received 1 week of initial treatment with dalteparin (5000 IU/d) before randomization	LMWH (dalteparin, 5000 IU/d for 5 weeks; administered by s.c. injection; compliance, 94.6%)	Matching placebo for 5 weeks (compliance with injections, 94.6%)	Incidence of DVT confirmed by unilateral phlebography after cast removal or compression ultrasonography where the phlebography failed
Lassen et al.*,* 2002 k^34^	Denmark (6 hospitals)	R, DB^a^	Adults (>18 y; median age, 47 y; male, 52%) Fracture or rupture of the Achilles tendon Conservative or surgically treated Outpatient (in most cases) N = 440					
	Within 4 d of injury Immobilization duration: 44 d^b^	Yes, approximately one‐third in each group received other LMWH for up to 4 d before randomization	LMWH (reviparin, 1750 IU/d for duration of cast immobilization; administered by s.c. injection; compliance, approximately 100%)	Matching placebo for duration of cast immobilization (compliance with injections, approximately 100%)	Incidence of DVT determined by unilateral venography after plaster cast removal (or earlier if clinical symptoms of thrombosis suspected)			
Selby et al*.,* 2015 8	Canada (13 hospitals)	R, DB^a^	Patients over 16 y (mean age, 49 y; male, 52%) fractures Surgically treated Outpatients N = 265	Within 72 h of injury Immobilization duration: 43 d^b^	No	LMWH (dalteparin, 5000 IU/d for 14 d; administered by s.c. injection; compliance, 90%)	Matching placebo for 14 d (compliance with injections, 92%)	Symptomatic VTE within 3 months after surgery or asymptomatic proximal DVT determined by bilateral Doppler ultrasound at end of treatment
van Adrichem et al*.,* 2017 36	The Netherlands (8 hospitals)	R, OL^a^	Adults (>18 y; mean age, 46 y; male, 49.9%) Fracture or soft‐tissue injury Conservative or surgically treated Outpatients N = 1519	NR Immobilization duration: 4.9 weeks^b^	No	LMWH (nadroparin, 2850 IU/d or dalteparin, [2500 IU/d for <100 kg or 5000 IU/d >100 kg] for duration of cast immobilization; administered by s.c. injection; compliance, 87%)	No treatment	Incidence of symptomatic VTE within 3 months after the procedure. DVT determined by abnormal compression ultrasound
Zheng et al.*,* 2017 6	China (3 hospitals)	R, DB^a^	Adults (>18 y; mean age, 47.8 y; male, 62.3%) Fracture of the ankle or foot Surgically treated Outpatients N = 814	Mean 3.3 d Immobilization duration: NR	No	LMWH (NR but given once daily for 14 d; administered by s.c. injection; compliance, NR)	Matching placebo for 14 d (compliance with injections, NR)	Incidence of VTE. DVT determined by bilateral Doppler ultrasound
Gehling et al.*,* 1998 27	Germany (1 hospital)	R	Patients over 16 y (mean age, 36 y; male, 49%) Fracture or soft‐tissue injury Management approach unclear (but majority appear to be surgically treated) Outpatients N = 287	NR Immobilization duration: NR	NR	LMWH (reviparin, 1750 IU/d administered by s.c. injection; compliance, NR)	Aspirin (1000 mg/d administered orally; compliance, NR)	Incidence of DVT determined by duplex sonography (all) or phlebography (if thrombosis suspected)
Bruntink et al.*,* 2017 (3‐arm study) 9	The Netherlands (7 hospitals)	R, SB^a^	Adults (>18 y; mean age, 47 y; male, 42%) Fracture of the ankle or foot Conservative treatment Outpatients N = 467	Within 72 h of injury Immobilization duration: 39.5 d^b^	No	LMWH nadroparin, 2850 IU/d for duration of cast immobilization; administered by s.c. injection; compliance approximately 100%)	1. Fondaparinux (2.5 mg/d for duration of cast immobilization; administered by s.c. injection; compliance, approximately 100%) 2. No treatment	Incidence of DVT determined by duplex sonography after the removal of the cast (or earlier if thrombosis was suspected)
Samama et al.*,* 2013 35	France, Russia, The Netherlands, Spain, Germany, Italy (93 centers)	R, OL^a^	Adults (>18 y; mean age, 46 y; male, 46.6%) Fracture or soft‐tissue injury Conservative treatment Outpatients N = 1349	Within 72 h of injury Immobilization duration: 33.7 d^b^	No	LMWH (nadroparin, 2850 IU/d for duration of cast immobilization; administered by s.c. injection; compliance, NR)	Fondaparinux (2.5 mg/d for duration of cast immobilization; administered by s.c. injection; compliance, NR)	Incidence of VTE. Compression ultrasonography and/or venography performed for suspected DVT after cast removal

Abbreviations: DB, double blind; DVT, deep vein thrombosis; LMWH, low molecular weight heparin; NR, not reported; OL, open label; R, randomized controlled trial; SB, single blind; s.c, subcutaneous; VTE, venous thromboembolism.

a Blinded outcome assessment.

b Means calculated from reported group means of intervention and comparator arms.

Five studies were open label with subjective screening outcomes (duplex sonography or phlebography on cast removal).^29^, ^30^, ^31^, ^35^, ^36^ Six studies used double blinding within the design.^6^, ^8^, ^28^, ^32^, ^33^, ^34^ Although all studies included adult patients with an isolated lower limb injury requiring temporary immobilization, there was wide variation in terms of injury type. Five studies included only patients with fractures,^6^, ^8^, ^9^, ^28^, ^32^ one of patients with Achilles tendon ruptures,^33^ and the remaining seven studies included patients with mixed pathology.^27^, ^29^, ^30^, ^31^, ^34^, ^35^, ^36^ Depending on the type of injury, the management of lower limb injury included conservative treatment,^9^, ^30^, ^31^, ^35^ surgical management,^6^, ^8^, ^28^, ^32^, ^33^ or both.^29^, ^34^, ^36^ In eight studies,^6^, ^8^, ^9^, ^28^, ^32^, ^33^, ^34^, ^35^ patients were recruited within 4 days of injury, whereas, in the remaining studies,^27^, ^29^, ^30^, ^31^, ^36^ the time to recruitment was not stated. The duration of immobilization ranged from 14 days^28^ to 44 days.^32^, ^34^ In two studies, all^32^ or some (approximately one‐third)^34^ patients first received prophylaxis before randomization; these studies were included because any final impact on outcome would likely take the form of reduction in VTE outcome events. In addition, the results of these trials remain relevant to the study question in light of current regimes suggesting prophylaxis should continue for the duration of immobilization (usually 4‐6 weeks).

### 3.3 Risk of bias within and across studies

**Figure 2:**
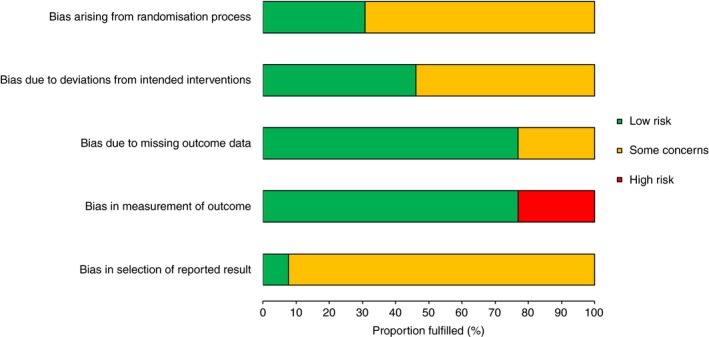
Risk of bias assessment graph: Review authors' judgments about each methodological quality item across all included studies

The overall methodological quality of the 13 included studies is summarized in Figure 2 and Table 2. Overall, risk of bias was present in all studies. Ten studies raised some concerns of bias.^6^, ^8^, ^9^, ^28^, ^29^, ^32^, ^33^, ^34^, ^35^, ^36^ The potential sources of bias most frequently identified included concerns about the randomization process (allocation concealment was not reported in nine studies),^6^, ^27^, ^28^, ^29^, ^30^, ^31^, ^32^, ^33^, ^34^ blinding (open‐label design)^9^, ^27^, ^29^, ^30^, ^31^, ^35^, ^36^, and analyses intentions (only one study provided sufficient information on selection of the reported result).^36^ High risk of bias was principally attributable to outcome assessment; in three open‐label studies, outcome assessment was performed on all patients with compression ultrasound and subsequent phlebography used to confirm positive sonographic findings. 27, 30, 31

**Table 2:** Risk of bias assessment summary: Review authors' judgments about each methodological quality item for each included study

Study	Bias arising from the randomization process	Bias from deviations from intended interventions	Bias due to missing outcome data	Bias in measurement of the outcome	Bias in selection of the reported result	Overall^a^
Goel et al.*,* 2009 28	Some concerns	Low	Low	Low	Some concerns	Some concerns
Jørgensen et al.*,* 2002 29	Some concerns	Some concerns	Low	Low	Some concerns	Some concerns
Kock et al.*,* 1995^30^	Some concerns	Some concerns	Low	High	Some concerns	High
Kujath et al.*,* 1993 31	Some concerns	Some concerns	Some concerns	High	Some concerns	High
Lapidus et al.*,* 2007a 32	Some concerns	Low	Low	Low	Some concerns	Some concerns
Lapidus et al.*,* 2007b 33	Some concerns	Low	Low	Low	Some concerns	Some concerns
Lassen et al.*,* 2002 34	Some concerns	Low	Low	Low	Some concerns	Some concerns
Selby et al.*,* 2015 8	Low	Low	Low	Low	Some concerns	Some concerns
van Adrichem et al.*,* 2017 36	Low	Some concerns	Low	Low	Low	Some concerns
Zheng et al.*,* 2017 6	Some concerns	Low	Some concerns	Low	Some concerns	Some concerns
Gehling et al.*,* 1998 27	Some concerns	Some concerns	Some concerns	High	Some concerns	High
Bruntink et al.*,* 2017 9	Low	Some concerns	Low	Low	Some concerns	Some concerns
Samama et al.*,* 2013 35	Low	Some concerns	Low	Low	Some concerns	Some concerns

a Overall risk of bias judgment (equal to the most severe level of bias found in any domain) was judged as: 1) Low risk of bias: the study is judged to be at low risk of bias for all domains for this result; 2) Some concerns: the study is judged to have some concerns of bias in at least one domain for this result; 3) High risk of bias: the study is judged to be at high risk of bias in at least one domain for this result or have some concerns for multiple domains in a way that substantially lowers confidence in the result.

### 3.4 Effects of interventions

Details of the total participant numbers in each analysis, event rates, and further key outcome results of the individual primary studies are provided in Table 3. All 13 studies reported outcomes for any VTE, PE, and major bleeding. The rate of any VTE in the control group ranged from 1.8% to 40.4%. The rate of PE in the control group was zero in eight studies and ranged from 0.7% to 2.1% in the other four. There was only one major bleeding event across all control groups.

**Table 3:** Summary of outcomes: PE and major bleeding

Author, year	Comparison	PE						Major bleeding					
		LMWH		Fondaparinux		Control		LMWH		Fondaparinux		Control	
		Events	Total	Events	Total	Events	Total	Events	Total	Events	Total	Events	Total
Goel et al.*,* 2009^28^	LMWH vs placebo	0	127 (0%)	‐	‐	0	111 (0%)	0	127 (0%)	‐	‐	0	111 (0%)
Jørgensen et al.*,* 2002^29^	LMWH vs no treatment	0	99 (0%)	‐	‐	0	106 (0%)	0	99 (0%)	‐	‐	0	106 (0%)
Kock et al.*,* 1995^30^	LMWH vs no treatment	0	176 (0%)	‐	‐	0	163 (0%)	0	176 (0%)	‐	‐	0	163 (0%)
Kujath et al.*,* 1993^31^	LMWH vs no treatment	0	126 (0%)	‐	‐	0	127 (0%)	0	126 (0%)	‐	‐	0	127 (0%)
Lapidus et al.*,* 2007a^32^	LMWH vs placebo	0	49 (0%)	‐	‐	0	47 (0%)	0	49 (0%)	‐	‐	0	47 (0%)
Lapidus et al.*,* 2007b^33^	LMWH vs placebo	0	117 (0%)	‐	‐	0	109 (0%)	0	117 (0%)	‐	‐	0	109 (0%)
Lassen et al.*,* 2002^34^	LMWH vs placebo	0	183 (0%)	‐	‐	2	188 (1.1%)	2	217 (0.9%)	‐	‐	1	221 (0.5%)
Selby et al.*,* 2015^8^	LMWH vs placebo	0	130 (0%)	‐	‐	1	128 (0.8%)	0	134 (0%)	‐	‐	0	131 (0%)
van Adrichem et al.*,* 2017 36	LMWH vs no treatment	4	719 (0.6%)	‐	‐	5	716 (0.7%)	0	719 (0%)	‐	‐	0	716 (0%)
Zheng et al.*,* 2017^6^	LMWH vs placebo	0	411 (0%)	‐	‐	0	403 (0%)	0	411 (0%)	‐	‐	0	403 (0%)
Gehling et al.*,* 1998^27^	LMWH vs aspirin	0	143 (0%)	‐	‐	0	144 (0%)	0	143 (0%)	‐	‐	0	144 (0%)
Bruntink et al.*,* 2017^9^	LMWH vs fondaparinux vs no treatment	0	92 (0%)	0	92 (0%)	2	94 (2.1%)	0	92 (0%)	0	92 (0%)	0	94 (0%)
Samama et al.*,* 2013^35^	LMWH vs fondaparinux	0	622 (0%)	2	621 (0.3%)	‐	‐	0	670 (0%)	1	674 (0.1%)	‐	‐

Abbreviations: DVT, deep vein thrombosis; LMWH, low molecular weight heparin; PE, pulmonary embolism.

**Figure 3:**
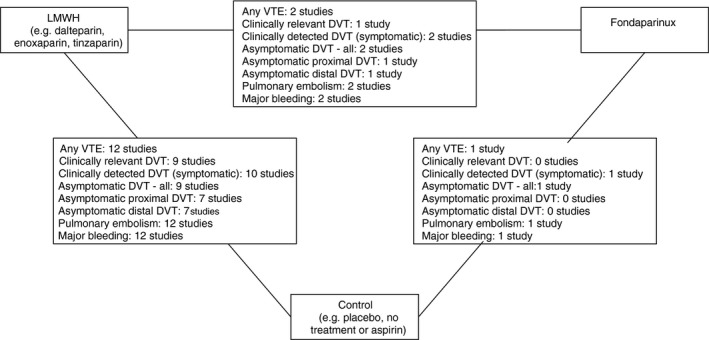
Network diagram of different pharmacological thromboprophylaxis interventions versus no thromboprophylaxis for preventing VTE^a,b^. DVT, deep vein thrombosis; LMWH, low molecular weight heparin; VTE, venous thromboembolism. ^a^The nodes are the interventions. The numbers against each outcome represent the number of times that each pair of interventions has been compared. There was one multi‐arm study comparing LMWH versus fondaparinux versus control. ^b^Diagrams for specific outcomes depends on the number of studies that provide data and the number of non‐zero event studies; not all outcomes involve feedback loops

NMA was undertaken to compare the effectiveness of two alternative forms of thromboprophylaxis (LMWH or fondaparinux) to no thromboprophylaxis (aspirin, placebo, or no treatment). Figure 3 presents the network of evidence. All 13 studies were included in the analysis and provided information on at least one of the outcomes being analyzed. A summary of the results of fixed effect and random effects NMA are provided in Table 4.

**Table 4:** Results of fixed effect and random effects NMA of different pharmacological thromboprophylaxis interventions versus no thromboprophylaxis

	Fixed effect odds ratio (95% CrI)	Random effects odds ratio (95% CrI)	Odds ratio (95% PrI)	Prob. Best
Clinically detected DVT (symptomatic):				
LMWH	0.45 (0.22‐0.89)	0.40 (0.12‐0.99)	0.41 (0.05‐2.31)	0.09
Fondaparinux	0.11 (0.01‐0.60)	0.10 (0.01‐0.94)	0.10 (0.00‐1.46)	0.91
None	‐	‐	‐	0.00
Asymptomatic DVT (proximal segment):				
LMWH	0.22 (0.05‐0.71)	0.21 (0.04‐0.82)	0.21 (0.02‐1.34)	0.63
Fondaparinux	0.29 (0.03‐2.35)	0.28 (0.02‐3.42)	0.28 (0.01‐4.49)	0.36
None	‐	‐	‐	0.01
Asymptomatic DVT (distal):				
LMWH	0.69 (0.47‐1.01)	0.69 (0.43‐1.12)	0.69 (0.29‐1.62)	0.00
Fondaparinux	0.11 (0.04‐0.27)	0.11 (0.03‐0.35)	0.11 (0.03‐0.42)	1.00
None	‐	‐	‐	0.00
Asymptomatic DVT (all):				
LMWH	0.57 (0.42‐0.77)	0.57 (0.39‐0.82)	0.57 (0.28‐1.12)	0.00
Fondaparinux	0.14 (0.07‐0.27)	0.14 (0.05‐0.31)	0.14 (0.05‐0.38)	1.00
None	‐	‐	‐	0.00
Pulmonary embolism:				
LMWH	0.30 (0.07‐0.96)	0.17 (0.01‐0.88)	0.18 (0.00‐1.79)	0.74
Fondaparinux	0.64 (0.05‐7.26)	0.47 (0.01‐9.54)	0.48 (0.01‐17.53)	0.25
None	‐	‐	‐	0.01
Major bleeding:				
LMWH	1.60 (0.14‐25.67)	1.45 (0.08‐32.17)	1.46 (0.06‐42.87)	0.37
Fondaparinux	14380 (0.48‐9.9E14)	8422 (0.32‐1.3E14)	8421 (0.29‐1.3E14)	0.03
None	‐	‐	‐	0.59
Clinically relevant DVT^a^				
LMWH	0.43 (0.22‐0.79)	0.40 (0.16‐0.85)	0.40 (0.07‐1.76)	0.22
Fondaparinux	0.25 (0.07‐0.82)	0.23 (0.03‐1.36)	0.23 (0.02‐ 2.11)	0.77
None	‐	‐	‐	0.01
Any VTE:				
LMWH	0.53 (0.41‐0.67)	0.52 (0.37‐0.71)	0.52 (0.23‐1.12)	0.00
Fondaparinux	0.14 (0.07‐0.25)	0.13 (0.05‐0.30)	0.13 (0.04‐0.39)	1.00
None	‐	‐	‐	0.00

Abbreviations: CrI, credible interval; PrI, predictive interval.

a Clinically relevant DVT was defined as the cumulative figure of any symptomatic OR asymptomatic proximal DVT.

#### 3.4.1 Clinically detected DVT (symptomatic)

Data were available from all 13 studies.^6^, ^8^, ^9^, ^27^, ^28^, ^29^, ^30^, ^31^, ^32^, ^33^, ^34^, ^35^, ^36^ The risk of clinically detected DVT (symptomatic) was lower in adult outpatients with lower limb immobilization who received LMWH (OR, 0.40; 95% CrI: 0.12‐0.99) and fondaparinux (OR, 0.10; 95% CrI: 0.01‐0.94) compared with control. Fondaparinux is likely to be the most effective treatment (probability of being the most effective = 0.91).

#### 3.4.2 Asymptomatic DVT: proximal segment

Data were available from eight studies.^6^, ^8^, ^27^, ^28^, ^29^, ^30^, ^33^, ^35^ The risk of asymptomatic DVT (proximal segment) was lower in adult outpatients with lower limb immobilization who received LMWH (OR, 0.21; 95% CrI: 0.04‐0.82) compared with control. A similar effect was found for fondaparinux, although the results were inconclusive (OR, 0.28; 95% CrI: 0.02‐3.42).

#### 3.4.3 Asymptomatic DVT: distal

Data were available from eight studies.^6^, ^8^, ^27^, ^28^, ^29^, ^30^, ^34^, ^35^ The risk of asymptomatic DVT (distal) was lower in adult outpatients with lower limb immobilization who received fondaparinux (OR, 0.11; 95% CrI: 0.03‐0.35) compared with control; fondaparinux is likely to be the most effective treatment (probability of being the most effective = 1.00). There was insufficient evidence of an effect of LMWH (OR, 0.69; 95% CrI: 0.43‐1.12) compared with control, although the effect favored treatment with LMWH.

#### 3.4.4 Asymptomatic DVT: all

Data were available from 10 studies.^6^, ^8^, ^9^, ^27^, ^28^, ^29^, ^30^, ^32^, ^34^, ^35^ The risk of asymptomatic DVT (all) was lower in adult outpatients with lower limb immobilization who received LMWH (OR, 0.57; 95% CrI: 0.39‐0.82) and fondaparinux (OR, 0.14; 95% CrI: 0.05‐0.31) compared with control. Fondaparinux is likely to be the most effective (probability of being the most effective = 1.00).

#### 3.4.5 Pulmonary embolism

Data were available from all 13 studies.^6^, ^8^, ^9^, ^27^, ^28^, ^29^, ^30^, ^31^, ^32^, ^33^, ^34^, ^35^, ^36^ The risk of PE was lower in adult outpatients with lower limb immobilization who received LMWH (OR, 0.17; 95% CrI: 0.01‐0.88) compared with control. A reduction in risk was also found for fondaparinux, although the results were inconclusive (OR, 0.47; 95% CrI: 0.01‐9.54).

#### 3.4.6 Any VTE

Data were available from all 13 studies.^6^, ^8^, ^9^, ^27^, ^28^, ^29^, ^30^, ^31^, ^32^, ^33^, ^34^, ^35^, ^36^ The risk of any VTE was lower in adult outpatients with lower limb immobilization who received LMWH (OR, 0.52; 95% CrI: 0.37‐0.71) and fondaparinux (OR, 0.13; 95% CrI: 0.05‐0.30) compared with no thromboprophylaxis. Fondaparinux is likely to be the most effective treatment (probability of being the most effective = 1.00). Although the results suggest that the true effects may vary according to study characteristics, the predictive distribution still favored fondaparinux relative to no prevention or placebo.

#### 3.4.7 Major bleeding

Data were available from all 13 studies, reporting major bleeding rates up to 0.9% with LMWH, 0.1% with fondaparinux, and 0.5% with control. 6, 8, 9, 27, 28, 29, 30, 31, 32, 33, 34, 35, 36 Major bleeding event rates across all included studies are highlighted in Table 3. With only four events across all studies, the effects of LMWH (OR, 1.45; 95% CrI: 0.08‐32.17) and fondaparinux on the risk of major bleeding were inconclusive.

#### 3.4.8 Compliance and adverse events

Compliance with study medication appeared generally good within trial participants; eight studies reported >90% compliance, two studies between 80% and 90%, and was unclear in three studies. A single open‐label study^9^ recorded reports of pain on injection in 1.4% of participants within the intervention group.

There were few reported adverse events in the treated patients. Subjective and composite overall adverse event rates ranged from 0% to 4.0% across individual studies with intervention, and 0% to 2.0% in control patients. Minor bleeding event rates varied from 0% to 10.5% in the LMWH intervention groups, 0% to 1.5% in the fondaparinux intervention groups, and 0% to 6.8% in the control groups. In the largest RCT to date,^36^ the most common adverse event (of infection) occurred at a similar rate between intervention and control groups (1.6% vs 2.0%, respectively). In four studies actively reporting the incidence of heparin‐induced thrombocytopenia, no cases were found.^8^, ^29^, ^37^, ^38^ No deaths in any study were deemed attributable to either VTE or the use of intervention.

### 3.5 Additional analyses

The results of the network meta‐regressions are detailed in Table S3. The analysis showed that no covariate improved model fits and therefore explained variation in treatment effects.

A sensitivity analysis excluding the three studies at high risk of bias is detailed in Table S4. This analysis generally had negligible impact on the estimates of treatment effect, but as expected, tended to increase uncertainty.

The effect of the type of thromboprophylactic agent used (certoparin, dalteparin, nadroparin, reviparin, and tinzaparin) was assessed using a separate NMA. This showed evidence to suggest that there were differences in the effects of the type of thromboprophylactic agent used, including between the different types of LMWH, with certoparin having the highest probability of the greatest effect on any VTE. These findings should be treated with caution, based on the heterogeneity between studies and the low event rates.

## 4 DISCUSSION

### 4.1 Summary of evidence

Our NMA shows that thromboprophylaxis with LMWH for patients with lower limb immobilization after injury approximately halves the odds of any VTE in these studies. Thromboprophylaxis with fondaparinux appears to have a greater effect on reducing the risk of DVT and is likely to be more effective than LMWH. Event rates for symptomatic DVT and PE in untreated patients were generally low across the studies, so an approximate halving of odds may result in a small absolute risk reduction.

Major bleeding is very uncommon, so the effect of thromboprophylaxis on major bleeding in this group is uncertain. Meta‐regression did not identify any reliable evidence of effect modification by key covariates.

### 4.2 Strengths and limitations

Our NMA synthesized data from 6857 participants in 13 randomized trials. This represents a large, methodologically robust data set across multiple settings used to simultaneously estimate of relative treatment effects.

Our analysis was inevitably limited by the primary data. The variety of settings and patient groups may be a strength, but generated evidence of heterogeneity of treatment effect across studies for many of the outcomes. Previous work has shown evidence of publication bias such that studies with nonsignificant or unfavorable results on this topic are perhaps less likely to be published, have a delay to publication, or involve selectively reporting outcomes.^39^ These issues have the potential to exaggerate any benefit to the intervention seen at NMA.

The studies were judged mainly to have low risk of bias or some concerns only for most quality criteria. However, three trials were judged as having a high risk of bias on the basis of outcome ascertainment being potentially subject to bias in an open‐label trial.^27^, ^30^, ^31^ This is particularly relevant to the issue of symptomatic VTE as an outcome. Several of these open‐label trials performed routine sonographic screening on removal of plaster cast, followed by clinical assessment. This methodology introduces a high risk of bias with symptomatic VTE outcomes; patients may have been influenced by the sonographer, or party to the ultrasound results before disclosing information on symptomatology. A sensitivity analysis excluding these studies generally had negligible impact on the estimates of treatment effect but, as expected, tended to increase uncertainty. This analysis does not take into account that several of the clinical events were likely not representative for events that would lead a patient to actively seek medical assistance (i.e., truly symptomatic events). This is reflected in the highly varying risks between studies found in Table 3. A further breakdown of symptomatic VTE outcomes is provided in Table S5 for transparency.

We included one trial^27^ that administered high‐dose aspirin to the control group, on the basis that at the time of review national UK guidelines on venous thromboembolism CG92^40^ and NG89^41^ did not consider aspirin or other antiplatelet agents to be appropriate for VTE prophylaxis in isolation.^42^, ^43^ If aspirin has a significant prophylactic effect, then this trial may underestimate the beneficial effect of additional thromboprophylaxis. As such, inclusion of this trial in the meta‐analysis would only confer bias toward a negative result.

The primary studies had a number of selection criteria that limit our ability to apply the findings to certain populations. Patients with a high risk of VTE (such as those with active cancer, thrombophilia, previous VTE, or pregnancy/puerperium) and those with an increased risk of bleeding were often excluded. The studies generally included patients with rigid immobilization rather than those with a degree of movement or a removable cast or splint, so the findings may only be applicable to those with full immobilization.

In addition, included studies range across a 25‐year period of publication, during which it is likely that management strategies have significantly evolved. Both immobilization and acute surgical intervention techniques have become less invasive over time, with early mobilization and enhanced recovery routinely promoted. There is also ongoing debate about the merits of thrombosis research using asymptomatic VTE as any form of outcome. Concerns include the use of variable criteria and assessment strategies to confirm disease and the impact of observer bias in unblinded studies using these outcomes.^44^, ^45^ Conversely, some authors highlight the evidence suggesting asymptomatic VTE to be a potential indicator of the relative risk of symptomatic VTE and even fatal PE.^46^ Thromboprophylaxis after lower limb injury has specific challenges in these areas, given the variation in management and the inevitable presence of leg symptoms at baseline injury (swelling and pain). To what degree do symptoms need to change to suggest a risk of symptomatic VTE to both the patient and clinician? Both these issues are perhaps reflected in the highly variable incidence of VTE across the included studies, ranging from 1.8% to 40.4%.^47^ We present our outcomes in this study stratified by symptomatic disease and anatomical location of VTE to address these issues.

The analysis included a substantial number of participants but the number of events for some outcomes were very low, or zero, including zero events in two arms of a study. As a consequence, not all studies provide estimates of relative treatment effect for all outcomes. We were unable to produce precise estimates of the effect of thromboprophylaxis upon major bleeding or estimate the effect of treatment on death. The low rate of bleeding provides some reassurance that thromboprophylaxis is not causing a clinically important rate of serious adverse outcome in this population but this may not be applicable to patients with a higher risk of bleeding. Other surrogate datasets, such as patients receiving thromboprophylaxis for knee arthroscopy can provide further relevant information on bleeding risk.^48^ This information could be used to support shared decision‐making. However, the population undergoing elective arthroscopy has key differences to our population of interest, including acute exposure to surgical bleeding risk, hospitalization, and the absence of blunt forced injury. As such, extrapolation of bleeding risk to conservatively managed ambulatory patients has significant caveats.

### 4.3 Comparison to previous research

Two systematic reviews have been recently published on the use of pharmacological thromboprophylaxis for patients with temporary immobilization resulting from acute injury. Hickey et al. included seven studies, focussing only on LMWH as an intervention and reporting an OR of 0.29 for the development of symptomatic DVT, with limited precision (95% CI 0.09‐0.95).^49^ In addition, they note a low major bleeding rate (0.1%) with LMWH. These findings are in keeping with the results of this study.

An updated Cochrane meta‐analysis by Zee et al.^50^ reported data from eight trials,^9^, ^29^, ^30^, ^31^, ^32^, ^33^, ^34^, ^36^ including 3680 participants that compared thromboprophylaxis with no treatment or placebo. They found that LMWH was associated with a significantly reduced risk of any DVT (OR, 0.45; 95% CI: 0.33‐0.61) and symptomatic VTE (OR, 0.40; 95% CI: 0.21‐0.76). Zee et al.^50^ excluded four trials that were included in our analysis (Goel et al.^28^, Selby et al.^8^, Gehling et al.,^27^ and Samama et al.^35^), whereas one additional trial was published after their updated meta‐analysis (Zheng et al.^6^). Two of the trials were excluded because they focused on operatively treated fractures rather than immobilization (Goel et al.^28^, Selby et al.^8^), one because the comparator was aspirin (Gehling et al.^27^) and one because the intervention was fondaparinux rather than LMWH (Samama et al.)*.* 35 The inclusion of these trials has ensured that our analysis is more comprehensive, but possibly at the expense of greater heterogeneity.

In addition, our study is also the first to perform network meta‐analysis (NMA) of different treatment options. NMA allows indirect comparison of interventions and facilitates assessment of benefits and harms for variable treatment options for a given clinical scenario. This methodology has recently been used by the World Health Organization to inform clinical guideline development, is considered to be a high level of evidence, and has specific advantages for VTE research in which multiple treatment options exist for a single pathology.^51^

### 4.4 Meaning of the study

Thromboprophylaxis in lower limb immobilization resulting from injury approximately halves the odds of any VTE and is associated with reductions in the risks of symptomatic DVT and PE.

The impact of this reduction in odds is likely to have variable clinical impact dependent on baseline risk. If baseline risk for symptomatic disease is consistently low across a population, then halving the odds may result in a low absolute risk reduction (ARR) and a high number needed to treat (NNT). This issue is demonstrated in Table 3, in which the summation of events for clinically relevant DVT results in a reduction from a 1.7% event rate (control) to a 1.0% event rate (LMWH). Many clinicians may consider this benefit too limited to justify the cost and potential adverse event profile of LMWH. However, assuming the relative treatment effect is consistent, a selected population with a higher baseline risk (identified through risk scoring or alternative method) would be expected to receive a larger proportional ARR and a resulting lower NNT, which may produce a different clinical decision. For this reason, single reported ARR and NNT derived from meta‐analysis have been reported as potentially misleading.

The evidence found was limited to LMWH and fondaparinux; it remains unclear whether these findings can be extrapolated to DOAC agents or other modalities. This is an important consideration because the absolute risks of clinically relevant VTE may vary across populations; patients who may not be willing to submit to the inconvenience of parenteral treatment to reduce a relatively small risk may be prepared to use oral therapy.

Within the meta‐regression analyses, we were unable to identify any evidence to directly support stratified thromboprophylaxis. We found no association between treatment effect and patient characteristics, type of injury, treatment method, or duration of prophylaxis. Several authors have recently suggested that selection of patients for thromboprophylaxis may be appropriate on the basis of an increased baseline risk^39^; it does not appear from our analysis adjusting for baseline risk that prophylaxis should be offered based on an expectation of greater effectiveness in any specific group. We did not assess risk factors at a patient level within this work and so cannot draw any conclusions on the merits of risk adjusted thromboprophylaxis.

### 4.5 The direction of future research

Although our findings suggest that thromboprophylaxis could reduce the rate of symptomatic events, further study of cost effectiveness is needed given the low absolute risk. In addition, stratified thromboprophylaxis may be able to select out patients at highest risk and maximize potential clinical and cost effectiveness. Several risk assessment models (RAM) have already been derived for use in this patient population, aiming to tailor thromboprophylaxis strategies at presumed high risk and limit financial costs, opportunity costs, and side effects.^12^, ^52^, ^53^ The current evidence base for RAMs is very limited, and estimates of sensitivity and specificity are subject to substantial uncertainty.^17^ Improving the evidence base for RAMs is a key research priority and external validation studies are urgently needed. In addition to dichotomous RAMs, individualized treatment could potentially be optimized by further adaptation in very high‐risk groups deemed to warrant thromboprophylaxis (e.g., higher dosing, longer duration). This latter question is yet to be addressed in the literature.

Oral medications could provide the benefits of thromboprophylaxis without the costs, inconvenience, and discomfort of injections. However, evidence of effectiveness in our review was related only to LMWH or fondaparinux. If further research identifies a high‐risk population that unequivocally benefit from thromboprophylaxis, the use of direct oral anticoagulants could potentially be compared with LMWH to assess differences in cost, clinical outcome, and patient satisfaction.

It is currently unclear whether people with limited lower limb immobilization (such as splints that allow some movement or removable splints or casts) carry similar risks of VTE to those with full immobilization. A study of this population could determine the risk of VTE and potentially identify patient‐level risk predictors.

## 5 CONCLUSIONS

Thromboprophylaxis for patients with lower limb immobilization after injury appears to be clinically effective, reducing the odds of symptomatic VTE. Given the low absolute risk of VTE in a broad population, individualized risk assessment and shared decision making may be optimal. This strategy requires further supporting research.

## CONFLICT OF INTERESTS

On behalf of all authors, I declare no known real or potential conflicts of interest to exist regarding this research article.

## AUTHOR CONTRIBUTIONS

D. Horner, A. Pandor, and S. Goodacre were responsible for identifying the research question, obtaining funding, and drafting of the initial protocol. J.W. Stevens was responsible for the statistical aspects. T. Nokes, K. de Wit, and J. Keenan provided clinical expertise throughout the project. D. Horner and A. Pandor were responsible for the drafting of this paper, although all authors provided comments on the drafts and read and approved the final version. D. Horner is the guarantor for the paper.
