# Supplementary material for: Pharmacological thromboprophylaxis to prevent venous thromboembolism in patients with temporary lower limb immobilization after injury: systematic review and network meta‐analysis
Source: J Thromb Haemost. 2019 Dec 1;18(2):422–38. doi: 10.1111/jth.14666 (PMC7028118; doi:10.1111/jth.14666)
Supplement: Supplementary file 1 [file JTH-18-422-s001.docx]

**Table S1: Literature search strategies for the review of pharmacological thromboprophylaxis for preventing VTE**

| **Database searched:** | **Ovid MEDLINE(R) Epub Ahead of Print, In-Process & Other Non-Indexed Citations, Ovid MEDLINE(R) Daily, Ovid MEDLINE and Versions(R)** |
| --- | --- |
| **Platform or provider used:** | **Ovid SP** |
| **Date of coverage:** | **1946 to April 2017** |
| **Search undertaken:** | **April 2017** |
|  |  |

1 thrombosis/ or exp venous thrombosis/

2 Venous Thromboembolism/ or Thromboembolism/

3 exp Pulmonary Embolism/

4 (thromboprophyla* or thrombus* or thrombotic* or thrombolic* or thromboemboli* or thrombos* or embol*).ti,ab,kw.

5 ((vein* or ven*) adj7 thromb*).ti,ab,kw.

6 (PE or DVT or VTE).ti,ab,kw.

7 1 or 2 or 3 or 4 or 5 or 6

8 exp Immobilization/

9 exp Mobility Limitation/

10 Splints/

11 Braces/

12 exp Casts, Surgical/

13 immobili*.mp. [mp=title, abstract, original title, name of substance word, subject heading word, keyword heading word, protocol supplementary concept word, rare disease supplementary concept word, unique identifier, synonyms]

14 brace*.mp. [mp=title, abstract, original title, name of substance word, subject heading word, keyword heading word, protocol supplementary concept word, rare disease supplementary concept word, unique identifier, synonyms]

15 splint*.mp. [mp=title, abstract, original title, name of substance word, subject heading word, keyword heading word, protocol supplementary concept word, rare disease supplementary concept word, unique identifier, synonyms]

16 plaster*.mp. [mp=title, abstract, original title, name of substance word, subject heading word, keyword heading word, protocol supplementary concept word, rare disease supplementary concept word, unique identifier, synonyms]

17 cast.mp. [mp=title, abstract, original title, name of substance word, subject heading word, keyword heading word, protocol supplementary concept word, rare disease supplementary concept word, unique identifier, synonyms]

18 (leg* or tibia* or fibula* or ankle*).mp. and (fracture*.hw. or su.fs.) and co.fs.

19 8 or 9 or 10 or 11 or 12 or 13 or 14 or 15 or 16 or 17 or 18

20 exp Heparin, Low-Molecular-Weight/

21 (heparin* or LMWH or nadroparin* or fraxiparin* or enoxaparin or Clexane or klexane or lovenox or dalteparin or Fragmin or ardeparin or normiflo or tinzaparin or logiparin or Innohep or certoparin or sandoparin or reviparin or clivarin* or danaproid or danaparoid or bemiparin or bioparin or Alphaparin or Troparin).mp. [mp=title, abstract, original title, name of substance word, subject heading word, keyword heading word, protocol supplementary concept word, rare disease supplementary concept word, unique identifier, synonyms]

22 (antixarin or ardeparin* or bemiparin* or Zibor or cy 222 or embolex or monoembolex or Mono-embolex or parnaparin* or "rd 11885" or tedelparin or Kabi-2165 or Kabi 2165).mp. [mp=title, abstract, original title, name of substance word, subject heading word, keyword heading word, protocol supplementary concept word, rare disease supplementary concept word, unique identifier, synonyms]

23 (emt-966 or emt-967 or pk-10169 or pk10169).mp. [mp=title, abstract, original title, name of substance word, subject heading word, keyword heading word, protocol supplementary concept word, rare disease supplementary concept word, unique identifier, synonyms]

24 (cy-216 or cy216 or seleparin* or tedegliparin or seleparin* or tedegliparin* or tedelparin or Boxol or Liquemine).mp. [mp=title, abstract, original title, name of substance word, subject heading word, keyword heading word, protocol supplementary concept word, rare disease supplementary concept word, unique identifier, synonyms]

25 fr-860.mp. [mp=title, abstract, original title, name of substance word, subject heading word, keyword heading word, protocol supplementary concept word, rare disease supplementary concept word, unique identifier, synonyms]

26 (wy90493 or wy-90493).mp. [mp=title, abstract, original title, name of substance word, subject heading word, keyword heading word, protocol supplementary concept word, rare disease supplementary concept word, unique identifier, synonyms]

27 (kb-101 or kb101 or lomoparan or orgaran).mp. [mp=title, abstract, original title, name of substance word, subject heading word, keyword heading word, protocol supplementary concept word, rare disease supplementary concept word, unique identifier, synonyms]

28 (parnaparin or fluxum or lohepa or lowhepa or "op 2123" or parvoparin).mp. [mp=title, abstract, original title, name of substance word, subject heading word, keyword heading word, protocol supplementary concept word, rare disease supplementary concept word, unique identifier, synonyms]

29 (AVE5026 or M118 or RO-14).mp. [mp=title, abstract, original title, name of substance word, subject heading word, keyword heading word, protocol supplementary concept word, rare disease supplementary concept word, unique identifier, synonyms]

30 20 or 21 or 22 or 23 or 24 or 25 or 26 or 27 or 28 or 29

31 (Rivaroxaban or xarelto or Apixaban or eliquis or Edoxaban or lixiana or Dabigatran or pradaxa or prazaxa or Praxibind or idaricuzimab).mp.

32 (novel oral anticoagulant* or novel oral anti-coagulant* or (new adj3 (anticoagulant* or anti-coagulant*))).mp.

33 (direct oral anticoagulant* or direct oral anti-coagulant*).mp. [mp=title, abstract, original title, name of substance word, subject heading word, keyword heading word, protocol supplementary concept word, rare disease supplementary concept word, unique identifier, synonyms]

34 (NOAC* or DOAC*).mp. [mp=title, abstract, original title, name of substance word, subject heading word, keyword heading word, protocol supplementary concept word, rare disease supplementary concept word, unique identifier, synonyms]

35 exp Aspirin/

36 (acetylsalicylic acid* or aspirin).mp.

37 31 or 32 or 33 or 34 or 35 or 36

38 7 and 19 and 30

39 limit 38 to yr="2013 -Current"

40 7 and 19 and 37

41 39 or 40

| **Databases searched:** | **Cochrane Database of Systematic Reviews, Cochrane Central Register of Controlled Trials, Database of Abstracts of Review of Effects, Health Technology Assessment Database and NHS Economic Evaluation Database** |
| --- | --- |
| **Platform or provider used:** | **Wiley Online** |
| **Date of coverage:** | **1898 to April 2017** |
| **Search undertaken:** | **April 2017** |
|  |  |

#1 MeSH descriptor: [Thrombosis] explode all trees

#2 MeSH descriptor: [Venous Thrombosis] explode all trees

#3 MeSH descriptor: [Thromboembolism] explode all trees

#4 MeSH descriptor: [Venous Thromboembolism] explode all trees

#5 MeSH descriptor: [Pulmonary Embolism] explode all trees

#6 (thromboprophyla* or thrombus* or thrombotic* or thrombolic* or thromboemboli* or thrombos* or embol*):ti,ab,kw (Word variations have been searched)

#7 ((vein* or ven*) near/7 thromb*):ti,ab,kw

#8 (PE or DVT or VTE):ti,ab,kw

#9 #1 or #2 or #3 or #4 or #5 or #6 or #7 or #8

#10 MeSH descriptor: [Immobilization] explode all trees

#11 MeSH descriptor: [Mobility Limitation] explode all trees

#12 MeSH descriptor: [Splints] explode all trees

#13 MeSH descriptor: [Braces] explode all trees

#14 MeSH descriptor: [Casts, Surgical] explode all trees

#15 (immobili* or brace* or splint* or plaster* or cast):ti,ab,kw

#16 ((leg* or tibia* or fibula* or ankle*) and (fracture* or surg*) and complication*):ti,ab,kw

#17 #10 or #11 or #12 or #13 or #14 or #15 or #16

#18 MeSH descriptor: [Heparin, Low-Molecular-Weight] explode all trees

#19 (heparin* or LMWH or nadroparin* or fraxiparin* or enoxaparin or Clexane or klexane or lovenox or dalteparin or Fragmin or ardeparin or normiflo or tinzaparin or logiparin or Innohep or certoparin or sandoparin or reviparin or clivarin* or danaproid or danaparoid or bemiparin or bioparin or Alphaparin or Troparin):ti,ab,kw

#20 (antixarin or ardeparin* or bemiparin* or Zibor or cy 222 or embolex or monoembolex or Mono-embolex or parnaparin* or "rd 11885" or tedelparin or Kabi-2165 or Kabi 2165):ti,ab,kw

#21 (emt-966 or emt-967 or pk-10169 or pk10169):ti,ab,kw

#22 fr-860:ti,ab,kw

#23 (wy90493 or wy-90493 or kb-101 or kb101 or lomoparan or orgaran or parnaparin or fluxum or lohepa or lowhepa or "op 2123" or parvoparin or AVE5026 or M118 or RO-14):ti,ab,kw

#24 #18 or #19 or #20 or #21 or #22 or #23

#25 #9 and #17 and #24 Publication Year from 2013 to 2017

#26 MeSH descriptor: [Anticoagulants] explode all trees

#27 (Rivaroxaban or xarelto or Apixaban or eliquis or Edoxaban or lixiana or Dabigatran or pradaxa or prazaxa or praxibind or idaricuzimab):ti,ab,kw

#28 (novel oral anticoagulant* or novel oral anti-coagulant* or (new near/3 (anticoagulant* or anti-coagulant*))):ti,ab,kw

#29 (direct oral anticoagulant* or direct oral anti-coagulant*):ti,ab,kw

#30 (NOAC* or DOAC*):ti,ab,kw

#31 MeSH descriptor: [Aspirin] explode all trees

#32 (acetylsalicylic acid* or aspirin):ti,ab,kw

#33 #26 or #27 or #28 or #29 or #30 or #31 or #32

#34 #9 and #17 and #33

#35 #25 or #34

| **Databases searched:** | **EMBASE** |
| --- | --- |
| **Platform or provider used:** | **Ovid SP** |
| **Date of coverage:** | **1974 to April 2017** |
| **Search undertaken:** | **April 2017** |
|  |  |

1 thrombosis/ or exp vein thrombosis/ or deep vein thrombosis/

2 Venous Thromboembolism/ or Thromboembolism/

3 exp lung embolism/

4 (thromboprophyla* or thrombus* or thrombotic* or thrombolic* or thromboemboli* or thrombos* or embol*).ti,ab,kw.

5 ((vein* or ven*) adj7 thromb*).ti,ab,kw.

6 (PE or DVT or VTE).ti,ab,kw.

7 1 or 2 or 3 or 4 or 5 or 6

8 exp Immobilization/

9 exp Mobility Limitation/

10 Splints/

11 Braces/

12 exp Casts, Surgical/

13 immobili*.mp. [mp=title, abstract, heading word, drug trade name, original title, device manufacturer, drug manufacturer, device trade name, keyword, floating subheading word]

14 brace*.mp. [mp=title, abstract, heading word, drug trade name, original title, device manufacturer, drug manufacturer, device trade name, keyword, floating subheading word]

15 splint*.mp. [mp=title, abstract, heading word, drug trade name, original title, device manufacturer, drug manufacturer, device trade name, keyword, floating subheading word]

16 plaster*.mp. [mp=title, abstract, heading word, drug trade name, original title, device manufacturer, drug manufacturer, device trade name, keyword, floating subheading word]

17 cast.mp. [mp=title, abstract, heading word, drug trade name, original title, device manufacturer, drug manufacturer, device trade name, keyword, floating subheading word]

18 (leg* or tibia* or fibula* or ankle*).mp. and (fracture*.hw. or su.fs.) and co.fs.

19 8 or 9 or 10 or 11 or 12 or 13 or 14 or 15 or 16 or 17

20 exp Heparin, Low-Molecular-Weight/

21 (heparin* or LMWH or nadroparin* or fraxiparin* or enoxaparin or Clexane or klexane or lovenox or dalteparin or Fragmin or ardeparin or normiflo or tinzaparin or logiparin or Innohep or certoparin or sandoparin or reviparin or clivarin* or danaproid or danaparoid or bemiparin or bioparin or Alphaparin or Troparin).mp. [mp=title, abstract, heading word, drug trade name, original title, device manufacturer, drug manufacturer, device trade name, keyword, floating subheading word]

22 (antixarin or ardeparin* or bemiparin* or Zibor or cy 222 or embolex or monoembolex or Mono-embolex or parnaparin* or "rd 11885" or tedelparin or Kabi-2165 or Kabi 2165).mp. [mp=title, abstract, heading word, drug trade name, original title, device manufacturer, drug manufacturer, device trade name, keyword, floating subheading word]

23 (emt-966 or emt-967 or pk-10169 or pk10169).mp. [mp=title, abstract, heading word, drug trade name, original title, device manufacturer, drug manufacturer, device trade name, keyword, floating subheading word]

24 (cy-216 or cy216 or seleparin* or tedegliparin or seleparin* or tedegliparin* or tedelparin or Boxol or Liquemine).mp. [mp=title, abstract, heading word, drug trade name, original title, device manufacturer, drug manufacturer, device trade name, keyword, floating subheading word]

25 fr-860.mp. [mp=title, abstract, heading word, drug trade name, original title, device manufacturer, drug manufacturer, device trade name, keyword, floating subheading word]

26 (wy90493 or wy-90493).mp. [mp=title, abstract, heading word, drug trade name, original title, device manufacturer, drug manufacturer, device trade name, keyword, floating subheading word]

27 (kb-101 or kb101 or lomoparan or orgaran).mp. [mp=title, abstract, heading word, drug trade name, original title, device manufacturer, drug manufacturer, device trade name, keyword, floating subheading word]

28 (parnaparin or fluxum or lohepa or lowhepa or "op 2123" or parvoparin).mp. [mp=title, abstract, heading word, drug trade name, original title, device manufacturer, drug manufacturer, device trade name, keyword, floating subheading word]

29 (AVE5026 or M118 or RO-14).mp. [mp=title, abstract, heading word, drug trade name, original title, device manufacturer, drug manufacturer, device trade name, keyword, floating subheading word]

30 20 or 21 or 22 or 23 or 24 or 25 or 26 or 27 or 28 or 29

31 (Rivaroxaban or xarelto or Apixaban or eliquis or Edoxaban or lixiana or Dabigatran or pradaxa or prazaxa or Praxibind or idaricuzimab).mp.

32 (novel oral anticoagulant* or novel oral anti-coagulant* or (new adj3 (anticoagulant* or anti-coagulant*))).mp.

33 (direct oral anticoagulant* or direct oral anti-coagulant*).mp. [mp=title, abstract, heading word, drug trade name, original title, device manufacturer, drug manufacturer, device trade name, keyword, floating subheading word]

34 (NOAC* or DOAC*).mp. [mp=title, abstract, heading word, drug trade name, original title, device manufacturer, drug manufacturer, device trade name, keyword, floating subheading word]

35 acetylsalicylic acid/

36 (acetylsalicylic acid* or aspirin).mp.

37 31 or 32 or 33 or 34 or 35 or 36

38 7 and 19 and 30

39 limit 38 to yr="2013 -Current"

40 7 and 19 and 37

41 39 or 40
