# Supplementary material for: Pharmacological thromboprophylaxis to prevent venous thromboembolism in patients with temporary lower limb immobilization after injury: systematic review and network meta‐analysis
Source: J Thromb Haemost. 2019 Dec 1;18(2):422–38. doi: 10.1111/jth.14666 (PMC7028118; doi:10.1111/jth.14666)
Supplement: Supplementary file 2 [file JTH-18-422-s002.docx]

**Table S2: Excluded studies with rationale - Review of pharmacological thromboprophylaxis for preventing VTE**

|  | **Author, year** | **Reason for exclusion** |
| --- | --- | --- |
|  | Ballester *et al.,* 2015^1^ | Not a randomised or controlled clinical trial (Letter to the Editor) |
|  | Blackwell *et al.,* 2017^2^ | Not a randomised or controlled clinical trial |
|  | Braithwaite *et al.,* 2016^3^ | Not a randomised or controlled clinical trial |
|  | Calder *et al.,* 2016^4^ | Systematic review |
|  | Chapelle *et al.,* 2014^5^ | Systematic review |
|  | Doggrell *et al.,* 2003^6^ | Review (non-systematic) |
|  | Ettema *et al.,* 2008^7^ | Systematic review |
|  | Griffiths *et al.,* 2012^8^ | Not a randomised or controlled clinical trial |
|  | Haque and Davies, 2015^9^ | Not a randomised or controlled clinical trial |
|  | Hickey *et al.,* 2016^10^ | Systematic review |
|  | Kaye *et al.,* 2015^11^ | Population: not isolated lower limb injury requiring temporary immobilisation |
|  | Kock *et al.,* 1993^12^ | Duplicate of included full text study: Kock *et al.,*1995^13^ |
|  | Little, 2016^14^ | Commentary |
|  | Mangwani *et al.,* 2015^15^ | Systematic review |
|  | Menakaya *et al.,* 2013^16^ | Not a randomised or controlled clinical trial |
|  | Metz *et al.,* 2009^17^ | Systematic review |
|  | Ramos *et al.,* 2008^18^ | Systematic review |
|  | Samama *et al.,* 2014^19^ | Duplicate of an included full text study: Samama *et al.,*2013^20^ |
|  | Samama *et al.,* 2013 (abstract)^21^ | Abstract of an included full text study: Samama *et al.,*2013^20^ |
|  | Samama *et al.,* 2013^22^ | Subgroup results of an included full text study: Samama *et al.,*2013^20^ |
|  | Spannagel and Kujath, 1993^23^ | Duplicate of included full text study: Kujath *et al.,*1993^24^ |
|  | Testroote *et al.,* 2014^25^ | Systematic review |
|  | Walenga *et al.,* 2014^26^ | Substudy of an included full text study (Lassen *et al.,*2002)^27^ - focus on biomarker evaluation |

**References:**

1. Ballester M, Saraiva De Sousa M, Ruiz-Ruiz J, et al. Venous thromboembolism in patients immobilised at home. *European Respiratory Journal* 2015; **45**(6): 1728-31.

2. Blackwell JR, Raval P, Quigley JP, Patel A, McBride D. Patient compliance with venous thromboembolism prophylaxis (VTE). *Journal of Clinical Orthopaedics and Trauma* 2017: no pagination.

3. Braithwaite I, Dunbar L, Eathorne A, Weatherall M, Beasley R. Venous thromboembolism rates in patients with lower limb immobilization after Achilles tendon injury are unchanged after the introduction of prophylactic aspirin: audit. *Journal of Thrombosis & Haemostasis* 2016; **14**(2): 331-5.

4. Calder JD, Freeman R, Domeij-Arverud E, van Dijk CN, Ackermann PW. Meta-analysis and suggested guidelines for prevention of venous thromboembolism (VTE) in foot and ankle surgery. *Knee Surg Sports Traumatol Arthrosc* 2016; **24**(4): 1409-20.

5. Chapelle C, Rosencher N, Jacques Zufferey P, et al. Prevention of venous thromboembolic events with low-molecular-weight heparin in the non-major orthopaedic setting: meta-analysis of randomized controlled trials. *Arthroscopy* 2014; **30**(8): 987-96.

6. Doggrell SA. Reviparin as prophylaxis for thromboembolism after leg injury and hip replacement. *Expert Opinion on Pharmacotherapy* 2003; **4**(2): 285-8.

7. Ettema HB, Kollen BJ, Verheyen CC, Buller HR. Prevention of venous thromboembolism in patients with immobilization of the lower extremities: a meta-analysis of randomized controlled trials. *Journal of Thrombosis and Haemostasis* 2008; **6**(7): 1093-8.

8. Griffiths JT, Matthews L, Pearce CJ, Calder JD. Incidence of venous thromboembolism in elective foot and ankle surgery with and without aspirin prophylaxis. *J Bone Joint Surg Br* 2012; **94**(2): 210-4.

9. Haque S, Davies MB. Oral thromboprophylaxis in patients with ankle fractures immobilized in a below the knee cast. *Journal of Foot & Ankle Surgery* 2015; **21**(4): 266-8.

10. Hickey BA, Watson U, Cleves A, et al. Does thromboprophylaxis reduce symptomatic venous thromboembolism in patients with below knee cast treatment for foot and ankle trauma? A systematic review and meta-analysis. *Foot and Ankle Surgery* 2016; **18**.

11. Kaye I, Patel D, Strauss E, et al. Prevention of Venous Thromboembolism after Arthroscopic Knee Surgery in a Low-Risk Population with the Use of Aspirin. A Randomized Trial. *Bulletin of the Hospital for Joint Disease (2013)* 2015; **73**(4): 243-8.

12. Kock HJ, Schmit-Neuerburg KP, Hanke J, et al. [Ambulatory prevention of thrombosis with low molecular weight heparin in plaster immobilization of the lower extremity].[German]. *Chirurg* 1993; **64**(6): 483-91.

13. Kock HJ, Schmit-Neuerburg KP, Hanke J, Rudofsky G, Hirche H. Thromboprophylaxis with low-molecular-weight heparin in outpatients with plaster-cast immobilisation of the leg. *Lancet* 1995; **346**(8973): 459-61.

14. Little MTM. Low-Molecular-Weight Heparin Did Not Differ from Placebo in Preventing Clinically Important Deep Venous Thrombosis After Surgical Repair of Leg Fracture. *J Bone Joint Surg Am* 2016; **98**(4): 316.

15. Mangwani J, Sheikh N, Cichero M, Williamson D. What is the evidence for chemical thromboprophylaxis in foot and ankle surgery? Systematic review of the English literature. *Foot* 2015; **25**(3): 173-8.

16. Menakaya CU, Boddice T, Malhotra R, et al. Outpatients thromboprophylaxis following lower limb immobilisation: an institution's experience. *European Orthopaedics and Traumatology* 2013; **5**(4): 335-9.

17. Metz R, Verleisdonk EJ, van der Heijden GJ. Insufficient Evidence for Routine Use of Thromboprophylaxis in Ambulatory Patients with an Isolated Lower Leg Injury Requiring Immobilization: Results of a Meta-Analysis. *Eur J Trauma Emerg Surg* 2009; **35**(2): 169-75.

18. Ramos J, Perrotta Carla, Badariotti G, Berenstein G. Interventions for preventing venous thromboembolism in adults undergoing knee arthroscopy. *Cochrane Database Syst Rev* 2008; (4): CD005259.

19. Samama CM, Lecoules N, Kierzek G, et al. Comparison of Fondaparinux with Low-Molecular-Weight Heparin for Venous Thromboembolism Prevention in Patients Requiring Rigid or Semi-Rigid Immobilization for Isolated Non-Surgical Below-Knee Injury. *Annales Francaises de Medecine d'Urgence* 2014; **4**(3): 153-66.

20. Samama CM, Lecoules N, Kierzek G, et al. Comparison of fondaparinux with low molecular weight heparin for venous thromboembolism prevention in patients requiring rigid or semi-rigid immobilization for isolated non-surgical below-knee injury. *Journal of Thrombosis & Haemostasis* 2013; **11**(10): 1833-43.

21. Samama CM, Riou B, Roy PM, Sautet A, Mismetti P. Prevention of venous thromboembolism after an isolated, non-surgical below-knee injury. Benefit/risk of fondaparinux vs. low molecular weight heparin: The FONDACAST study. *J Thromb Haemost* 2013; **11**: 5.

22. Samama CM, Riou B, Roy PM, Sautet A, Mismetti P, Van Der Veen A. Subgroup analysis of the FONDACAST study comparing fondaparinux to low-molecular-weight heparin for the prevention of venous thromboembolism after an isolated, non-surgical below-knee injury. *Journal of Thrombosis and Haemostasis* 2013; **11**: 88-9.

23. Spannagel U, Kujath P. Low molecular weight heparin for the prevention of thromboembolism in outpatients immobilized by plaster cast. *Semin Thromb Hemost* 1993; **19**(Suppl. 1): 131-41.

24. Kujath P, Spannagel U, Habscheid W. Incidence and prophylaxis of deep venous thrombosis in outpatients with injury of the lower limb. *Haemostasis* 1993; **23**(Suppl. 1): 20-6.

25. Testroote M, Stigter WA, Janssen L, Janzing HM. Low molecular weight heparin for prevention of venous thromboembolism in patients with lower-leg immobilization. *Cochrane Database Syst Rev* 2014; (4): CD006681.

26. Walenga JM, Kaiser PC, Prechel MM, et al. Sustained release of tissue factor following thrombosis of lower limb trauma. *Clinical & Applied Thrombosis/Hemostasis* 2014; **20**(7): 678-86.

27. Lassen M, Borris L, Nakov R. Use of the low-molecular-weight heparin reviparin to prevent deep-vein thrombosis after leg injury requiring immobilization. *N Engl J Med* 2002; **347**(10): 726-30.
