# Supplementary material for: Pharmacological thromboprophylaxis to prevent venous thromboembolism in patients with temporary lower limb immobilization after injury: systematic review and network meta‐analysis
Source: J Thromb Haemost. 2019 Dec 1;18(2):422–38. doi: 10.1111/jth.14666 (PMC7028118; doi:10.1111/jth.14666)
Supplement: Supplementary file 3 [file JTH-18-422-s003.docx]

**Table S3: Details of the network meta-regressions**

We assessed the following potential treatment effect modifiers: (1) Population characteristics (proportion male, baseline risk of VTE); (2) Type of injury (fractures, Achilles tendon rupture, other soft tissue injury); (3) Treatment of injury (surgical versus conservative, above versus below knee immobilisation); (4) Thromboprophylactic agent used; (5) Duration of thromboprophylaxis.

For each outcome of interest, except for major bleeding, a network meta-regression of the potential treatment effect modifiers 1-3 and 5 was performed separately for each covariate. The analyses were performed by centring the covariates at their mean values of the reference treatment (i.e. placebo, no treatment and aspirin) to improve mixing of the Markov chain Monte Carlo chains. In each model, the regression parameter was given a normally distributed prior distribution with mean 0 and variance 1000 i.e. N(0, 1000). Only Lassen *et al*., 2002^1^ and Samama *et al*., 2013^2^ provided information about major bleeding. Consequently, a meta-regression of major bleeding was not performed.

The analysis of the effect of the baseline risk of VTE was performed with respect to the true baseline rather than the sample estimate of the baseline in order to avoid regression to the mean. Assessing the effect simultaneously of three types of injury would have involved a model with two covariates to estimate in relatively sparse datasets; in practice, only the effect of fractures versus other type of injury was assessed. The effect of the management of the injury using a surgical or conservative approach, and the method of immobilisation whether above or below the knee, were assessed separately. Several studies (i.e. Kujath *et al.*, 1993,^3^ Samama *et al*., 2013,^2^ Selby *et al*., 2015,^4^ Van Adrichem *et al*., 2017^5^ and Zheng *et al*., 2017)^6^ provided insufficient information to allow a classification of whether the immobilisation was above or below the knee; these studies were excluded from the meta-regression.

In a network meta-regression, three assumptions can be made about the interaction between a covariate and each treatment effect: 1) Independent, treatment-specific interaction terms for each pair of treatments, 2) Exchangeable, treatment-specific but related interaction terms, and 3) Identical interaction effects for all treatments. Given the relative sparseness of the data, the analysis was performed only by assuming identical interaction terms for all treatments.

The effect of the type of thromboprophylactic agent used (i.e. dalteparin, tinzaparin, certoparin nadroparin, reviparin) was assessed using a separate NMA. Van Adrichem *et al*., 2017^5^ allowed nadroparin or dalteparin to be used according to the preference of each hospital and no further information was provided as to what was actually used. No information was available on the LMWH used in the Zheng *et al*., 2017 study.^6^ The Van Adrichem *et al*., 2017^5^ and Zheng *et al*., 2017^6^ studies were excluded from the analysis.

The unadjusted and covariate adjusted models were compared using the deviance information criterion. Models fitted to a particular dataset that have lower deviance information criterion values provide the best predictions. However, differences in deviance information criterion values less than five are generally not important and the simpler model is generally preferred irrespective of the estimated effect of individual parameters. In addition, deviation information criterion values can only be compared for models applied to the same datasets. Consequently, it is not possible to compare unadjusted and adjusted models with respect to the effect of immobilisation above or below the knee, or the type of thrompboprophylactic agent used because the analyses make use of data from different studies.

We were unable to use data from all the studies in all the analyses. Gehling *et al*., 1998^7^ and Goel *et al*., 2009^8^ had no asymptomatic DVT proximal events and consequently provided no information with which to estimate treatment effects or the effects of potential treatment effect modifiers. Jorgensen *et al*., 2002,^9^ Goel *et al*., 2009,^8^ Bruntink *et al*., 2017^10^ and Zheng *et al*., 2017^6^ had no clinically detected DVT events and consequently provided no information with which to estimate treatment effects or the effects of potential treatment effect modifiers. Goel *et al*., 2009^8^ had no clinically relevant DVT events and consequently provided no information with which to estimate treatment effects or the effects of potential treatment effect modifiers. Only Lassen *et al*., 2002,^1^ Samama *et al*., 2013,^2^ Selby *et al*., 2015,^4^ Bruntink *et al*., 2017^10^ and Van Adrichem *et al*., 2017^5^ had pulmonary embolisms; all other studies provided no information with which to estimate treatment effects or the effects of potential treatment effect modifiers.

The tables below show the results of the network meta-regression for all potential effect modifiers except thromboprophylactic agent used. Results according to the proportion of patients with immobilisation below the knee should be treated with caution as the model is fitted to a subset of the data and it is not possible to compare the fit of this model with the unadjusted model. The adjusted deviance information criterion (DIC) suggested that no covariate improved the model fits.

**Table S4a: Network meta-regression for all potential effect modifiers except thromboprophylactic agent used**

| **Covariate** | No Treatment^1^ | Adjusted  DIC | Regression Parameter |
| --- | --- | --- | --- |
| **Any VTE (**Unadjusted DIC=157.6) | | | |
| Proportion male | 56 | 159.1 | 0.007 (-0.029, 0.043) |
| Baseline VTE (%; logit) | 0.10 (-2.214) | 159.5 | -0.024 (-0.358, 0.299) |
| Type of injury: Proportion fractures | 68 | 158.9 | 0.003 (-0.007, 0.015) |
| Treatment of injury: Proportion surgery | 63 | 153.6 | 0.009 (0.002, 0.018) |
| Treatment of injury: Proportion below knee | 91 | NA | -0.001 (-0.043, 0.041) |
| Duration of thromboprophylaxis (Days) | 28 | 159.2 | 0.005 (-0.021, 0.033) |
| **Asymptomatic DVT (All)** (unadjusted DIC=113.9) | | | |
| Proportion male | 54 | 115.8 | -0.005 (-0.064, 0.053) |
| Baseline VTE (%; logit) | 0.12 (-2.039) | 172.1 | -0.003 (-0.452, 0.445) |
| Type of injury: Proportion fractures | 85 | 114.6 | 0.015 (-0.011, 0.050) |
| Treatment of injury: Proportion surgery | 71 | 112.1 | 0.012 (-0.000, 0.026) |
| Treatment of injury: Proportion below knee | 90 | NA | -0.005 (-0.045, 0.034) |
| Duration of thromboprophylaxis (Days) | 27 | 115.6 | -0.005 (-0.035, 0.025) |
| **Asymptomatic DVT (Distal)** (Unadjusted DIC=84.4) | | | |
| Proportion male | 56 | 85.6 | -0.040 (-0.153, 0.059) |
| Baseline VTE (%; logit) | 0.10 (-2.249) | 128.8 | -0.172 (-0.879, 0.510) |
| Type of injury: Proportion fractures | 81 | 84.7 | 0.022 (-0.011, 0.070) |
| Treatment of injury: Proportion surgery | 77 | 85.1 | 0.013 (-0.009, 0.044) |
| Treatment of injury: Proportion below knee | 85 | NA | -0.003 (-0.057, 0.050) |
| Duration of thromboprophylaxis (Days) | 23 | 86.2 | -0.005 (-0.045, 0.038) |
| **Asymptomatic DVT (Proximal)**  (Unadjusted DIC=44.8) |  |  |  |
| Proportion male | 60 | 46.6 | -0.018 (-0.205, 0.148) |
| Baseline VTE (%; logit) | 0.11 (-2.141) | 89.3 | -0.060 (-1.467, 1.123) |
| Type of injury: Proportion fractures | 69 | 46.5 | 0.003 (-0.032, 0.045) |
| Treatment of injury: Proportion surgery | 84 | 43.9 | 0.900 (0.045, 2.650) |
| Treatment of injury: Proportion below knee | 87 | NA | 1.289 (-0.128, 4.068) |
| Duration of thromboprophylaxis (Days) | 23 | 46.5 | -0.007 (-0.146, 0.111) |
| **Clinically Detected (DVT)** (Unadjusted DIC=64.4) | | | |
| Proportion male | 53 | 63.8 | -0.209 (-0.685, 0.056) |
| Baseline VTE (%; logit) | 0.10 (-2.229) | 127.0 | -0.514 (-1.505, 0.303) |
| Type of injury: Proportion fractures | 85 | 62.6 | 0.080 (0.003, 0.267) |
| Treatment of injury: Proportion surgery | 65 | 64.9 | 0.007 (-0.017, 0.041) |
| Treatment of injury: Proportion below knee | 90 | NA | 1.247 (0.101, 4.133) |
| Duration of thromboprophylaxis (Days) | 28 | 65.5 | -0.018 (-0.119, 0.076) |
| **Clinically Relevant (DVT)** (Unadjusted DIC=80.9) | | | |
| Proportion male | 58 | 81.0 | -0.062 (-0.176, 0.029) |
| Baseline VTE (%; logit) | 0.10 (-2.229) | 137.3 | -0.267 (-0.983, 0.377) |
| Type of injury: Proportion fractures | 75 | 81.1 | 0.017 (-0.008, 0.052) |
| Treatment of injury: Proportion surgery | 77 | 82.1 | 0.002 (-0.017, 0.027) |
| Treatment of injury: Proportion below knee | 88 | NA | 2.056 (0.128, 7.393) |
| Duration of thromboprophylaxis (Days) | 27 | 82.2 | 0.010 (-0.063, 0.082) |
| **Pulmonary Embolism** (Unadjusted DIC=38.2) | | | |
| Proportion male | 56 | 37.7 | 0.308 (-0.269, 1.062) |
| Baseline VTE (%; logit) | 0.09 (-2.277) | 104.2 | -15.190 (-36.670, -3.094) |
| Type of injury: Proportion fractures | 74 | 38.3 | -0.058 (-0.309, 0.191) |
| Treatment of injury: Proportion surgery | 63 | 38.8 | -0.057 (-0.263, 0.028) |
| Treatment of injury: Proportion below knee | 91 | NA | NE |
| Duration of thromboprophylaxis (Days) | 28 | 38.9 | 0.060 (-0.212, 0.488) |
| DIC, Deviance information criterion; NE: Not estimable  ^1^ Value used to centre the meta-regression | | | |

**Table S4b** shows the results according to the type of thromboprophylactic agent used. These findings should be treated with caution as the model is fitted to a subset of the data and it is not possible to compare the fit of this model with the original model. In the case of an asymptomatic DVT (Distal) outcome, the Samama *et al*., 2013 study^2^ is not connected; consequently, it is not possible to estimate the effects of nadroparin or fondaparinux on an asymptomatic DVT (Distal) outcome. In the case of an asymptomatic DVT (proximal segment) outcome, the Samama *et al*., 2013^2^ is not connected and the Gehling *et al.,* 1998 study^7^ had no events; consequently, it is not possible to estimate the effects of nadroparin, reviparin or fondaparinux on an asymptomatic DVT (proximal segment) outcome. In the case of a clinically detected (symptomatic) outcome, the Jorgensen *et al*., 2002^9^ and Bruntink *et al*, 2007^10^ studies had no events and provide no information about relative treatment effect; in addition, the Samama *et al*, 2013 study^2^ is only connected in the network via the nadroparin and fondaparinux arms of the Bruntink *et al*, 2007 study;^10^ consequently, it is not possible to estimate the effect of nadroparin, tinzaparin or fondaparinux on a clinically detected (symptomatic) outcome. In the case of a clinically relevant DVT outcome, the Samama *et al*., 2013 study^2^ is not connected; consequently, it is not possible to estimate the effects of nadroparin or fondaparinux on a clinically relevant DVT outcome.

There was evidence to suggest that there were differences in the effects of the type of thromboprophylactic agent used, including between the different types of LMWH, with certoparin having the highest probability of the greatest effect on any VTE. However, this is based on the effect of certoparin being used in one study (Kock *et al* 1995)^11^ so it is not possible to draw any reliable conclusions.

**Table S4b: Random effects NMA of different pharmacological thromboprophylaxis interventions versus no thromboprophylaxis**

|  | Odds ratio (95% CrI) | Odds ratio (95% PrI) | Probability best |
| --- | --- | --- | --- |
| **Clinically detected DVT (symptomatic):** | | | |
| Dalteparin | 0.38 (0.05, 2.60) | 0.38 (0.03, 5.28) | 0.01 |
| Tinzaparin | NE | NE | NE |
| Certoparin | 7.2E-10 (3.6E-31, 0.09) | 7.0E-10 (3.5E-31, 0.11) | 0.98 |
| Nadroparin | NE | NE | NE |
| Reviparin | 0.35 (0.03, 2,71) | 0.35 (0.02, 4.99) | 0.01 |
| Fondaparinux | NE | NE | NE |
| None | - | - | 0.00 |
| **Asymptomatic DVT (Proximal segment):** | | | |
| Dalteparin | 0.43 (0.04, 3.26) | 0.42 (0.03, 5.23) | 0.00 |
| Tinzaparin | 6.4E-10 (1.0E-29, 0.66) | 6.1E-10 (1.0E-29, 0.69) | 0.51 |
| Certoparin | 5.8E-10 (1.5E-32, 0.59) | 5.8E-10 (1.5E-32, 0.65) | 0.49 |
| Nadroparin | NE | NE | NE |
| Reviparin | NE | NE | NE |
| Fondaparinux | NE | NE | NE |
| None | - | - | 0.01 |
| **Asymptomatic DVT (Distal):** | | | |
| Dalteparin | 0.79 (0.25, 3.13) | 0.79 (0.15, 5.36) | 0.00 |
| Tinzaparin | 0.58 (0.13, 2.48) | 0.58 (0.08, 3.96) | 0.01 |
| Certoparin | 1.4E-10 (2.5E-30, 0.20) | 1.5E-10 (0.292.6E-30, 0.22) | 0.99 |
| Nadroparin | NE | NE | NE |
| Reviparin | 0.81 (0.29, 2.41) | 0.80 (0.16, 4.40) | 0.00 |
| Fondaparinux | NE | NE | NE |
| None | - | - | 0.00 |
| **Asymptomatic DVT (All):** | | | |
| Dalteparin | 0.72 (0.35, 1.59) | 0.72 (0.23, 2.54) | 0.00 |
| Tinzaparin | 0.54 (0.16, 1.84) | 0.54 (0.11, 2.54) | 0.00 |
| Certoparin | 3.5E-10 (4.7E-31, 0.13) | 3.5E-10 (4.6E-31, 0.13) | 0.96 |
| Nadroparin | 0.23 (0.04, 1.06) | 0.23 (0.03, 1.31) | 0.00 |
| Reviparin | 0.66 (0.29, 1.70) | 0.66 (0.20, 2.63) | 0.00 |
| Fondaparinux | 0.06 (0.01, 0.33) | 0.06 (0.01, 0.40) | 0.04 |
| None | - | - | 0.00 |
| **Pulmonary embolism:** | | | |
| Deltaparin | 4.2E-10 (9.0E-31, 0.43) | 4.2E-10 (9,3E-31, 0.47) | 0.27 |
| Tinzaparin | NE | NE | NE |
| Certoparin | NE | NE | NE |
| Nadroparin | 2.8E-13 (8.8E-28, 1.6E-3) | 2.7E-13 (8.7E-28, 1.9E-3) | 0.48 |
| Reviparin | 6.5E-10 (1.6E-27, 0.26) | 6.4E-10 (1.4E-27, 0.28) | 0.25 |
| Fondaparinux | 1.6E-6 (5.9E-15, 62) | 1.6E-6 (5.3E-15, 0.70) | 0.00 |
| None | - | - | 0.01 |
| **Major bleeding:** | | | |
| Deltaparin | NE | NE | NE |
| Tinzaparin | NE | NE | NE |
| Certoparin | NE | NE | NE |
| Nadroparin | NE | NE | NE |
| Reviparin | NE | NE | NE |
| Fondaparinux | NE | NE | NE |
| None | - | - | NE |
| **Clinically relevant DVT:** | | | |
| Deltaparin | 0.40 (0.10, 1.46) | 0.40 (0.06, 2.52) | 0.00 |
| Tinzaparin | 3.4E-10 (7.5E-32, 0.67) | 3.3E-10 (7.0E-32, 0.70) | 0.46 |
| Certoparin | 1.3E-11 (1.9E-31, 0.05) | 1.3E-11 (1.8E-31, 0.06) | 0.54 |
| Nadroparin | NE | NE | NE |
| Reviparin | 2.35 (0.14, 92.18) | 2.35 (0.11, 112.10) | 0.00 |
| Fondaparinux | 0.23 (0.03, 1.36) | 0.23 (0.02, 2.11) | 0.00 |
| None | - | - | 0.01 |
| **Any VTE:** | | | |
| Dalteparin | 0.69 (0.40, 1.23) | 0.68 (0.27, 1.83) | 0.00 |
| Tinzaparin | 0.54 (0.17, 1.61) | 0.54 (0.13, 2.95) | 0.00 |
| Certoparin | 8.5E-12 (9.9E-29, 0.02) | 0.528.6E-12 (9.8E-29, 0.02) | 0.99 |
| Nadroparin | 0.22 (0.08, 0.54) | 0.22 (0.06, 0.69) | 0.00 |
| Reviparin | 0.63 (0.31, 1.42) | 0.62 (0.23, 1.97) | 0.00 |
| Fondaparinux | 0.06 (0.02, 0.19) | 0.06 (0.01, 0.24) | 0.01 |
| None | - | - | 0.00 |
| CrI, credible interval; PrI, predictive interval; NE, Not estimable | | | |

The meta-regression used in this assessment was a between-study comparison and lacks information with which to estimate parameters compared to a within-study comparison using patient data. In addition, comparisons between treatments involving zero events within a study provide no information about the relative treatment effect or the relationship between a potential treatment effect modifier and treatment effect. The between-study meta-regression has the potential to suffer from the ecological fallacy such that the estimate of the relationship between a potential treatment effect modifier and treatment effect in a between-study comparison may be qualitatively different to the relationship between the treatment effect modifier and treatment effect within studies. Potential treatment effect modifiers were assessed separately whereas they could be affecting treatment effect simultaneously; there is insufficient information with which to estimate potential treatment effect modifiers simultaneously. An adjusted model that is indistinguishable from an unadjusted model may reflect a lack of evidence rather than a lack of a relationship between potential treatment effect modifier and treatment. Estimates of the regression parameter should be interpreted with caution when they indicate evidence of a relationship between a potential treatment effect modifier and treatment but the adjusted model is indistinguishable from the unadjusted model.

There was insufficient evidence to suggest that any of the potential treatment effect modifiers defined in the protocol affected the treatment effect.

There was evidence to suggest that there were differences in the effects of the type of thromboprophylactic agent used, including between the different types of LMWH.

References:

1. Lassen M, Borris L, Nakov R. Use of the low-molecular-weight heparin reviparin to prevent deep-vein thrombosis after leg injury requiring immobilization. *N Engl J Med* 2002; **347**(10): 726-30.

2. Samama CM, Lecoules N, Kierzek G, et al. Comparison of fondaparinux with low molecular weight heparin for venous thromboembolism prevention in patients requiring rigid or semi-rigid immobilization for isolated non-surgical below-knee injury. *Journal of Thrombosis & Haemostasis* 2013; **11**(10): 1833-43.

3. Kujath P, Spannagel U, Habscheid W. Incidence and prophylaxis of deep venous thrombosis in outpatients with injury of the lower limb. *Haemostasis* 1993; **23**(Suppl. 1): 20-6.

4. Selby R, Geerts WH, Kreder HJ, et al. A double-blind, randomized controlled trial of the prevention of clinically important venous thromboembolism after isolated lower leg fractures. *Journal of Orthopaedic Trauma* 2015; **29**(5): 224-30.

5. van Adrichem RA, Nemeth B, Algra A, et al. Thromboprophylaxis after Knee Arthroscopy and Lower-Leg Casting. *N Engl J Med* 2017; **376**(6): 515-25.

6. Zheng X, Li D-Y, Wangyang Y, et al. Effect of Chemical Thromboprophylaxis on the Rate of Venous Thromboembolism after Treatment of Foot and Ankle Fractures. *Foot & Ankle International* 2017; **37**(11): 1218-24.

7. Gehling H, Giannadakis K, Lefering R, Hessmann M, Achenbach S, Gotzen L. [Prospective randomized pilot study of ambulatory prevention of thromboembolism. 2 times 500 mg aspirin (ASS) vs. clivarin 1750 (NMH)]. *Unfallchirurg* 1998; **101**(1): 42-9.

8. Goel D, Buckley R, deVries G, Abelseth G, Ni A, Gray R. Prophylaxis of deep-vein thrombosis in fractures below the knee: a prospective randomised controlled trial. *J Bone Joint Surg Br* 2009; **91**(3): 388-94.

9. Jørgensen PS, Warming T, Hansen K, et al. Low molecular weight heparin (Innohep) as thromboprophylaxis in outpatients with a plaster cast: a venografic controlled study. *Thromb Res* 2002; **105**(6): 477-80.

10. Bruntink MM, Groutars YM, Schipper IB, et al. Nadroparin or fondaparinux versus no thromboprophylaxis in patients immobilised in a below-knee plaster cast (PROTECT): A randomised controlled trial. *Injury* 2017; **48**(4): 936-40.

11. Kock HJ, Schmit-Neuerburg KP, Hanke J, Rudofsky G, Hirche H. Thromboprophylaxis with low-molecular-weight heparin in outpatients with plaster-cast immobilisation of the leg. *Lancet* 1995; **346**(8973): 459-61.
