# Supplementary material for: Pharmacological thromboprophylaxis to prevent venous thromboembolism in patients with temporary lower limb immobilization after injury: systematic review and network meta‐analysis
Source: J Thromb Haemost. 2019 Dec 1;18(2):422–38. doi: 10.1111/jth.14666 (PMC7028118; doi:10.1111/jth.14666)
Supplement: Supplementary file 4 [file JTH-18-422-s004.docx]

**Table S4: Sensitivity analysis results of random effects NMA of different pharmacological thromboprophylaxis interventions versus no thromboprophylaxis**

|  | **Random Effects**  **Odds ratio (95% CrI)**  **Original** | **Random Effects**  **Odds ratio (95% CrI)**  **Sensitivity Analysis** | |
| --- | --- | --- | --- |
| **Clinically detected DVT (symptomatic):** | | |  |
| LMWH | 0.40 (0.12, 0.99) | 0.42 (0.11, 1.15) | |
| Fondaparinux | 0.10 (0.01, 0.94) | 0.10 (0.01, 0.95) | |
| **Asymptomatic DVT (Proximal segment):** | | | |
| LMWH | 0.21 (0.04, 0.82) | 0.23 (0.04, 0.98) | |
| Fondaparinux | 0.28 (0.02, 3.42) | 0.32 (0.02, 4.30) | |
| **Asymptomatic DVT (Distal):** | | | |
| LMWH | 0.69 (0.43, 1.12) | 0.67 (0.40, 1.17) | |
| Fondaparinux | 0.11 ( 0.03, 0.35) | 0.11 ( 0.03, 0.37) | |
| **Asymptomatic DVT (All):** | | | |
| LMWH | 0.57 (0.39, 0.82) | 0.55 (0.37, 0.81) | |
| Fondaparinux | 0.14 (0.05, 0.31) | 0.13 (0.05, 0.31) | |
| **Pulmonary embolism:** | | | |
| LMWH | 0.17 (0.01, 0.88) | 0.18 (0.01, 0.89) | |
| Fondaparinux | 0.47 (0.01, 9.54) | 0.48 (0.01, 10.16) | |
| **Major bleeding:** | | | |
| LMWH | 1.45 (0.08, 32.17) | 1.70 (0.09, 38.66) | |
| Fondaparinux | 8422 (0.32, 1.3E14) | 23890 (0.43, 2.0E14) | |
| **Clinically relevant DVT^a^** | | | |
| LMWH | 0.40 (0.16, 0.85) | 0.42 (0.16, 0.94) | |
| Fondaparinux | 0.23 (0.03, 1.36) | 0.25 (0.04, 1.34) | |
| **Any VTE:** | | | |
| LMWH | 0.52 (0.37, 0.71) | 0.56 (0.40, 0.77) | |
| Fondaparinux | 0.13 (0.05, 0.30) | 0.14 (0.06, 0.31) | |
| CrI, credible interval  ^a^ Clinically relevant DVT was defined as the cumulative figure of any symptomatic OR asymptomatic proximal DVT | | | |
