# Supplementary material for: Pharmacological thromboprophylaxis to prevent venous thromboembolism in patients with temporary lower limb immobilization after injury: systematic review and network meta‐analysis
Source: J Thromb Haemost. 2019 Dec 1;18(2):422–38. doi: 10.1111/jth.14666 (PMC7028118; doi:10.1111/jth.14666)
Supplement: Supplementary file 5 [file JTH-18-422-s005.docx]

| Author, year |  |  |  |  |
| --- | --- | --- | --- | --- |
|  |  |  |  |  |
|  | Total clinically relevant events | **DVT Diagnosed during US screening assessment and within routine follow up protocol** | Acute symptomatic DVT diagnosed outside of routine follow up protocol | Acute Diagnosis of Pulmonary Embolism |
| Goel *et al.,* 2009 | 0 (Control)  0 (Intervention) | 0  0 | 0  0 | 0  0 |
| Jørgensen *et al.,* 2002 | 1 (Control)  0 (Intervention) | 1 (100%)  0 | 0  0 | 0  0 |
| Kock *et al.,* 1995 | 5 (Control)  0 (Intervention) | 5 (100%)  0 | 0  0 | 0  0 |
| Kujath *et al.,* 1993 | 0 (Control)  0 (Intervention) | 0  0 | 0  0 | 0  0 |
| Lapidus *et al.,* 2007a | 3 (Control)  1 (Intervention) | 3 (100%)  1 | 0  0 | 0  0 |
| Lapidus *et al.,* 2007b | 6 (Control)  2 (Intervention) | 0  0 | 6 (100%)  2 | 0  0 |
| Lassen *et al.,* 2002 | 12 (Control)  3 (Intervention) | 6 (50%)  3 | 4 (33.3%)  0 | 2 (16.7%)  0 |
| Selby *et al.,* 2015 | 3 (Control)  2 (Intervention) | 1 (33.3%)  1 | 1 (33.3%)  1 | 1 (33.3%)  0 |
| van Adrichem *et al.,* 2017 | 14 (Control)  11 (Intervention) | 0  0 | 9 (64.3%)  7 | 5 (35.7%)  4 |
| Zheng *et al.,* 2017 | 6 (Control)  1 (Intervention) | 6 (100%)  1 | 0  0 | 0  0 |
| Gehling *et al.,* 1998 | 1 (Control)  2 (Intervention) | 1 (100%)  2 | 0  0 | 0  0 |
| Bruntink *et al.,* 2017 | 16 (Control)  0 (Intervention) | 14 (87.5%)  0 | 0  0 | 2 (12.5%)  0 |
| Samama *et al.,* 2013 | 0 (Control)  18 (Intervention) | 0  16 (88.9%) | 0  0 | 0  2 (11.1%) |

US – Ultrasound

DVT – Deep Vein Thrombosis

PE – Pulmonary Embolism
